# Evolutionary History of the *Rhinolophus pusillus* Species Complex Reveals Phylogeographic Diversification Across the East Asian Mainland–Island System

**DOI:** 10.3390/ani16172669

**Published:** 2026-08-25

**Authors:** Haijian Sun, Guangjian Zhu, Keqi Lu, Guimei He, Panyu Hua

**Affiliations:** 1 School of Ecological and Environmental Sciences, East China Normal University, Shanghai 200062, China; hjsunbio@gmail.com (H.S.); 51283903024@stu.ecnu.edu.cn (K.L.); 2 School of Life Sciences, East China Normal University, Shanghai 200062, China; gjzhu@bio.ecnu.edu.cn

**Keywords:** *Rhinolophus pusillus*, *Rhinolophus monoceros*, phylogeography, mitonuclear discordance, demographic expansion, Quaternary refugia

## Abstract

Widespread species complexes often contain hidden evolutionary diversity shaped by dispersal, isolation and climatic change. Here, we aimed to characterize geographic genetic structure within the *Rhinolophus pusillus* species complex, assess the concordance among genetic, morphological and acoustic patterns and the evolutionary distinctiveness of the Taiwan-endemic *R. monoceros*, and determine whether Quaternary changes in habitat suitability were geographically consistent with the inferred phylogeographic and demographic history. To address these objectives, we analyzed 378 individuals from 70 localities and integrated mitochondrial sequences, nuclear loci, microsatellite genotypes, external phenotypic traits comprising forearm length, body mass, forearm mass index and echolocation resting frequency, demographic inference, ancestral-area reconstruction and ecological niche modelling. Mitochondrial analyses identified five geographically structured lineages within *R. pusillus*, corresponding broadly to South China, East China, Central China, Hainan Island and Southwest China, while *R. monoceros* formed a closely related but distinctive Taiwan lineage. South China showed the highest mitochondrial diversity, occupied a central position in haplotype networks and was repeatedly inferred as an important ancestral and refugial region. Divergence-time estimates placed the early diversification of the complex in the Middle Pleistocene, followed by demographic expansion in several lineages during the Late Pleistocene. In contrast, nuclear loci and microsatellites revealed extensive allele sharing and weaker geographic subdivision, suggesting incomplete lineage sorting and historical connectivity. Our results indicate that the *R. pusillus* complex represents an ongoing diversification system shaped by Quaternary climatic fluctuations, sea-level changes and regional isolation, highlighting the value of integrative phylogeography for conserving hidden evolutionary diversity in widespread bats.

## Simple Summary

The *Rhinolophus pusillus* species complex is widely distributed across East Asia and includes several geographically isolated or taxonomically debated forms. Because morphology, echolocation calls and genetic markers have previously given partly conflicting results, its evolutionary history has remained unclear. In this study, we combined mitochondrial DNA, nuclear loci, microsatellites, morphology, acoustic data, demographic analyses, ancestral-area reconstruction and ecological niche modelling using range-wide samples from mainland China, Hainan and Taiwan. Our results show that *R. pusillus* contains several geographically structured mitochondrial lineages, while the Taiwan endemic *R. monoceros* represents a closely related but distinctive island lineage. South China appears to have been an important refugial and ancestral region, whereas Hainan, Taiwan and Southwest China experienced stronger historical isolation. Nuclear markers showed weaker differentiation, suggesting incomplete lineage sorting and historical connectivity. These findings indicate that the complex is still undergoing diversification and that several regional populations deserve conservation attention.

## 1. Introduction

Widespread species frequently harbour substantial cryptic diversity, providing valuable opportunities to investigate how historical dispersal, geographic isolation, and environmental change drive lineage diversification across heterogeneous landscapes [1,2]. Consequently, resolving the evolutionary history of widespread species complexes has become a central objective in evolutionary biology, biogeography, and integrative taxonomy. Recent advances in molecular systematics and integrative taxonomy have shown that combining independent lines of genetic, morphological, acoustic, and ecological evidence is essential for identifying cryptic evolutionary lineages and delimiting species boundaries [1]. Among mammals, horseshoe bats (*Rhinolophus*) represent one of the most species-rich and evolutionarily diverse genera, currently comprising more than 110 recognized extant species distributed throughout the Old World [3]. Recent phylogenomic and macroevolutionary analyses further suggest that repeated dispersal, ecological divergence, and Quaternary environmental change have been major drivers of diversification within the genus [4], whereas integrative taxonomic studies continue to reveal substantial cryptic diversity and previously unrecognized evolutionary lineages [5]. Together, these findings indicate that evolutionary relationships remain incompletely resolved in several widespread *Rhinolophus* species complexes, highlighting the need for comprehensive range-wide phylogeographic studies to reconstruct evolutionary history and identify the processes responsible for lineage diversification.

In this study, we use the term “*Rhinolophus pusillus* species complex” to refer to the focal assemblage comprising nominal *R. pusillus* from mainland China and Hainan Island together with the Taiwan-endemic nominal species *R. Monoceros*. Among East Asian horseshoe bats, the *Rhinolophus pusillus* species complex provides an exceptional model for investigating lineage diversification across mainland–island systems. The complex occupies one of the broadest geographic ranges within the genus, extending from continental East and Southeast Asia to Hainan and Taiwan Islands, where repeated cycles of land-bridge formation, geographic isolation, and climatic oscillation during the Quaternary created diverse opportunities for population divergence and secondary contact. Despite this biogeographic complexity, the evolutionary history of the complex remains poorly resolved. Multiple subspecies have been proposed throughout its distribution, yet studies based on morphology, echolocation call frequency, and mitochondrial DNA have frequently produced conflicting hypotheses regarding species limits and phylogenetic relationships [6,7,8,9,10,11]. These inconsistencies largely reflect the combined effects of morphological conservatism, overlapping acoustic traits, incomplete geographic sampling, and the limited resolution of single-marker datasets. Consequently, a comprehensive range-wide analysis integrating multilocus genetic, phenotypic, and biogeographic evidence is required to reconstruct the evolutionary history of the *R. pusillus* species complex and to identify the historical processes responsible for its diversification.

The least horseshoe bat, *Rhinolophus pusillus*, is one of the most widely distributed members of the genus in East Asia and constitutes the core lineage of the *R. pusillus* species complex. Its distribution extends across continental East and Southeast Asia and several continental shelf islands, including Hainan and Taiwan, providing an exceptional natural system for investigating how Quaternary climatic oscillations, sea-level fluctuations, and geographic isolation have shaped lineage diversification across mainland–island environments [2,12,13]. Despite its broad distribution, the evolutionary history of the complex remains contentious. Up to nine subspecies have been recognized based primarily on morphology and geographic distribution [7,14], yet their evolutionary relationships have remained uncertain. In one of the first comprehensive integrative studies of the complex, Li et al. [8] combined mitochondrial cytochrome *b (Cyt b)* sequences with echolocation frequency and forearm length and concluded that *R. pusillus*, the Taiwan endemic *R. monoceros*, and the Japanese endemic *R. cornutus* were conspecific, while also questioning the taxonomic validity of the Hainan subspecies *R. p. parcus*. These findings challenged the traditional taxonomic framework of the complex and established a long-standing debate regarding species boundaries and the evolutionary origin of the island lineages.

Despite considerable progress over the past two decades, the evolutionary relationships within the *Rhinolophus pusillus* species complex remain incompletely resolved because different lines of evidence have yielded conflicting evolutionary hypotheses. Morphological analyses supported the recognition of *R. pusillus*, *R. monoceros*, and *R. cornutus* as distinct species, although they failed to distinguish the currently recognized subspecies within *R. pusillus* [10]. Conversely, mitochondrial analyses suggested a close evolutionary affinity among the three nominal species but relied exclusively on a single maternally inherited marker, limiting their ability to distinguish recent divergence, incomplete lineage sorting, and historical introgression [8]. Subsequent phylogenetic analyses based primarily on mitochondrial DNA recovered additional evolutionary lineages within the species group but did not fully resolve relationships among the principal taxa or evaluate demographic processes across the entire geographic range [11]. Collectively, these studies demonstrate that current uncertainties arise not only from conflicting morphological and molecular evidence, but also from incomplete geographic sampling, the absence of multilocus nuclear data, and the lack of an integrative phylogeographic framework capable of simultaneously evaluating evolutionary history, lineage diversification, and historical population dynamics.

To resolve these outstanding questions, we conducted a comprehensive range-wide investigation of the *Rhinolophus pusillus* species complex across East Asia by integrating multilocus genetic data, previously published morphological and acoustic datasets, ecological niche modelling, and historical demographic inference. Specifically, we investigated geographic diversification within *R. pusillus* across mainland China and Hainan Island and evaluated its evolutionary relationship with the Taiwan-endemic species *R. monoceros*. We aimed to (1) characterize geographic genetic structure within *R. pusillus* using mitochondrial sequences, nuclear loci and microsatellite genotypes; (2) evaluate whether geographic differentiation was concordant across genetic, acoustic and morphological datasets and assess the distinctiveness of *R. monoceros* relative to *R. pusillus*; and (3) determine whether broad-scale changes in potential habitat suitability during the Quaternary were geographically consistent with the inferred phylogeographic and demographic history of the focal mainland–island system. The ecological niche modelling was conducted at the level of the focal *R. pusillus–R. monoceros* assemblage rather than as a test of species-specific niche differentiation. Because *R. monoceros* represents the Taiwan-endemic lineage central to the evolutionary history examined here, its occurrence records were included to characterize potential changes in habitat availability and connectivity across the full geographic extent of the complex.

## 2. Materials and Methods

### 2.1. Sampling and Phenotypic Analyses

Resting frequency (RF), body size and flight-related morphological traits are closely associated with foraging performance and ecological differentiation in insectivorous bats. To examine phenotypic variation across the *R. pusillus* and *R. monoceros*, we compared acoustic and morphological traits among populations throughout their distributional range.

**Figure 1:**
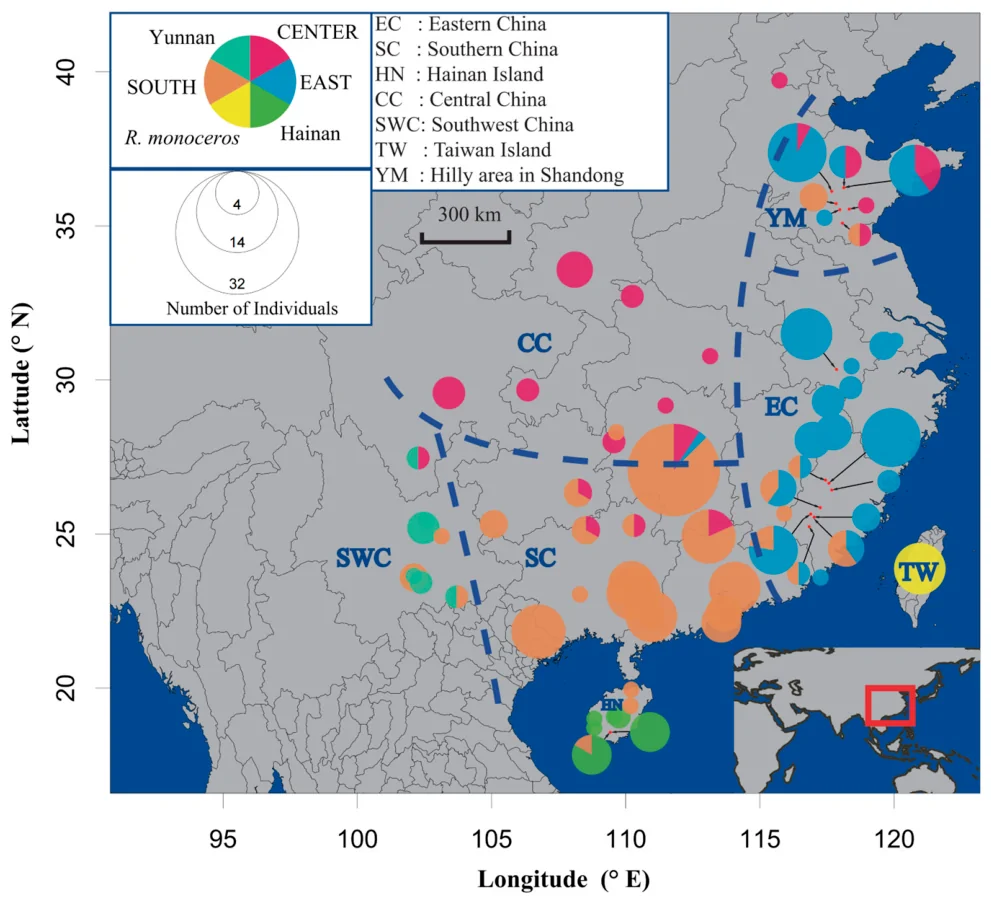
Geographic distribution, sampling localities and mitochondrial lineages of *R. pusillus* and *R. monoceros*. Sampling localities are shown as pie charts, with colours representing the major mitochondrial lineages recovered from the Bayesian phylogenetic analysis. The size of each pie chart is proportional to the number of sampled individuals at that locality. Dashed blue lines delimit the seven geographic groups used in this study: EC, East China; SC, South China; HN, Hainan Island; CC, Central China; SWC, Southwest China; YM, Yimeng Mountains, Shandong Province; and TW, Taiwan Island. The Taiwan group corresponds to *R. monoceros*, whereas the remaining groups correspond to *R. pusillus*. Red box in the inset indicates the location of the study area.

Field sampling was conducted from 2006 to 2017, primarily during May to October. Bats were captured using mist nets at 70 localities across East Asia (Figure 1). A wing-membrane biopsy was collected from each of 378 live individuals, after which the bats were released at the capture sites. Tissue samples were preserved in 95% ethanol and stored at −20 °C until DNA extraction. Resting frequency, forearm length (FA) and body mass were measured following Zhang et al. [15]. To maximize geographic coverage, previously published acoustic and morphological datasets [9,16,17,18], together with field records collected since 2002, were incorporated into subsequent analyses.

Body condition was estimated using the forearm mass index (FMI), calculated as body mass divided by forearm length squared [19]. Because direct wing-area measurements were unavailable, FMI was used as a comparative index of relative body condition and flight-related morphology among geographic populations of *R. pusillus*. However, because wing morphology and body proportions may differ among species, FMI was not used for direct interspecific comparison between *R. pusillus* and *R. monoceros*.

Individuals were assigned a priori to seven geographic population groups solely according to their sampling localities: East China (EC), South China (SC), Central China (CC), Hainan Island (HN), Southwest China (SWC; Yunnan and southern Sichuan), the Yimeng Mountains in Shandong Province (YM), and Taiwan Island (TW) (Figure 1). Morphological traits, resting frequency and molecular data were subsequently used to characterize and compare differentiation among these geographically defined groups. The Taiwan group corresponds to *R. monoceros*, whereas all remaining groups correspond to *R. pusillus*.

### 2.2. DNA Sequencing and Microsatellite Genotyping

Genomic DNA was extracted from wing membrane biopsies using DNeasy Tissue Kits (Qiagen, Hilden, Germany) according to the manufacturer’s instructions. Three mitochondrial loci were amplified and sequenced, including partial *Cyt b*, NADH dehydrogenase subunit 1 (ND1), and the mitochondrial control region (D-loop), which included the hypervariable region I.

To complement the mitochondrial dataset, four independent nuclear loci were sequenced: chromodomain helicase DNA-binding protein 1 (Chd1), the short-wavelength-sensitive opsin gene (SWS1), the thyrotropin intron (THY), and X-linked ubiquitin-specific protease 9 (USP9x). PCR amplification followed previously published protocols and primer sequences are provided in Table S1 [8,16,20,21,22,23]. Samples showing heterozygous sites, identified as double peaks in sequencing chromatograms, were phased using PHASE v2.1 [24].

To place the *R. pusillus* species complex in a broader phylogenetic context and reassess its taxonomic status, all publicly available sequences representing members of the *pusillus* group, closely related taxa of the *rouxii* group, and representative *Rhinolophus* species used as outgroups were incorporated into the analyses (Table S2). Newly generated sequences were aligned using CLUSTAL X v1.83 [25], followed by manual inspection and editing in MEGA v6 [26].

To investigate contemporary population structure, 11 polymorphic nuclear microsatellite loci (A10A, A4, E11A, PD3, PD9, PH30, PH69A, PH57, PH78, H3 and PH96) were genotyped in 337 individuals of *R. pusillus* and *R. monoceros* [27]. These mitochondrial, nuclear sequence and microsatellite markers have been widely used in phylogenetic and population genetic studies of *Rhinolophus* and provide complementary information across different evolutionary timescales [8,16,22,28,29].

### 2.3. Genetic Diversity, Phylogenetic Reconstruction and Population Structure

Haplotype number (*h*), haplotype diversity (Hd), nucleotide diversity (π), and the number of polymorphic sites were calculated for each mitochondrial marker using DnaSP v5.10 [30]. To place the genetic diversity of the *R. pusillus* species complex into a broader evolutionary context, these estimates were compared with published values reported for other bat species, with particular emphasis on geographically isolated taxa within *Rhinolophus*.

To reconstruct phylogenetic relationships within the *R. pusillus* species complex and evaluate the evolutionary distinctiveness of geographic lineages, phylogenetic analyses were performed independently for *Cyt b*, D-loop, ND1, the concatenated mitochondrial dataset, and each nuclear locus. Bayesian inference (BI) was conducted in MrBayes v3.2.6 [31], whereas maximum-likelihood (ML) analyses were performed in RAxML v8.2.4 [32]. The best-fitting nucleotide substitution model for each dataset was selected according to the corrected Akaike Information Criterion (AICc) in jModelTest v0.1.1 [33]. For BI analyses, two independent Markov chain Monte Carlo (MCMC) runs, each comprising four chains, were performed for 20 million generations, with trees sampled every 100 generations. The first 25% of sampled trees were discarded as burn-in after convergence had been confirmed.

Maximum-likelihood analyses employed the GTR+GAMMA substitution model with the rapid hill-climbing algorithm. For each dataset, 200 independent searches were performed from random starting trees rather than from the default maximum-parsimony starting tree, following recommendations for datasets of comparable size [32]. Branch support was assessed using 1000 bootstrap replicates.

Because bifurcating phylogenetic trees may not adequately represent genealogical relationships among closely related haplotypes [34,35], haplotype networks were reconstructed for each genetic marker using the statistical parsimony algorithm of TCS [36,37] implemented in PopART [38].

Population genetic structure based on microsatellite data was inferred using STRUCTURE v2.3.4 [39]. Analyses were conducted assuming K = 2–6. Replicate runs showing high similarity, defined by a symmetric similarity coefficient ≥0.8, were identified and merged using CLUMPP v1.1.2 [40]. Consensus ancestry coefficients were visualized in DISTRUCT v1.1 [41], and the most likely number of genetic clusters was determined using Structure Harvester v0.6.94 [42].

For the microsatellite dataset, linkage disequilibrium, departures from Hardy–Weinberg equilibrium (HWE), and potential genotyping artefacts were assessed using standard quality-control procedures, with significance corrected for multiple testing where appropriate. Rare-allele excess and scoring errors were examined in GenAlEx v6.5 [43], recent bottlenecks were tested under the stepwise mutation model in BOTTLENECK [44], and potential FST outlier loci were identified using LOSITAN [45]. Loci identified as outliers were excluded from subsequent population genetic analyses where appropriate.

To quantify hierarchical genetic differentiation and evaluate the geographic population structure inferred above, analyses of molecular variance (AMOVA) were performed in Arlequin v3.5 [46]. Genetic variation was partitioned among geographic groups, among populations within groups, and within populations. Statistical significance was assessed using 20,000 permutations. Pairwise ΦST values were additionally calculated for both the *Cyt b* and D-loop datasets.

### 2.4. Demographic Analyses

To estimate the time to the most recent common ancestor (TMRCA) of the major lineages, Bayesian molecular dating analyses were performed using the *Cyt b* dataset in BEAST v1.4.7 [47]. The GTR substitution model was selected for the *Cyt b* dataset according to the corrected Akaike Information Criterion in jModelTest. A maximum-likelihood molecular-clock test conducted in MEGA v6 did not reject the molecular-clock hypothesis (*p* > 0.05). Therefore, following previous studies [35,48,49], a strict molecular clock with a fixed substitution rate of 0.02 substitutions site^−1^ Myr^−1^ and a constant-size coalescent prior were applied. Two independent MCMC runs of 20 million generations were conducted, with samples recorded every 1000 generations and the first 10% discarded as burn-in. Convergence and effective sample sizes were assessed in Tracer v1.7, and the two runs were combined after stationarity had been confirmed [50].

Historical demographic changes were first evaluated using mismatch distribution analyses based on the *Cyt b* dataset. Pairwise nucleotide difference distributions were generated in DnaSP v5.10 and Arlequin v3.5 for the major geographic populations and mitochondrial clades [30,46]. In Arlequin, the fit of the observed mismatch distribution to a sudden demographic expansion model was assessed using the sum of squared deviations (SSD) and Harpending’s raggedness index (r), with 10,000 bootstrap replicates. In DnaSP, mismatch distributions were evaluated under both constant population size and population growth–decline models. For populations showing unimodal mismatch distributions consistent with demographic expansion, the timing of expansion was estimated according to τ = 2ut, where τ is the estimated expansion parameter and u = μgk, with μ = 0.02 substitutions site^−1^ Myr^−1^, g = 2 years and k = 1070 bp. Generation time was assumed to be two years based on our field observations and published estimates for *Rhinolophus* species [28,29,51], including *R. monoceros* [16].

Historical changes in effective population size were further evaluated using neutrality tests. Tajima’s D, Fu’s FS, and Ramos–Onsins and Rozas’ R2 statistics were calculated in DnaSP and Arlequin. Statistical significance was assessed using 10,000 coalescent simulations. Following previous studies [52,53,54], significant departures from neutrality were interpreted together with mismatch distributions as evidence of historical demographic change.

To further reconstruct historical changes in effective population size through time, Bayesian skyline plot (BSP) analyses were performed in BEAST. Analyses followed the settings of Lin et al. [55], with MCMC chains run for 50 million generations. Demographic trajectories were summarized from the posterior distribution after adequate MCMC convergence had been confirmed.

### 2.5. Biogeographic Analyses and Ecological Niche Modelling

To infer the historical biogeography of the *R. pusillus* species complex, ancestral distributions were reconstructed using both statistical dispersal–vicariance analysis (S-DIVA) and Bayesian binary MCMC (BBM) implemented in RASP v3.02 [56]. These complementary approaches were employed to account for uncertainty in ancestral-area reconstruction, particularly because the study area includes both the continental mainland and the island systems of Hainan and Taiwan. Based on the phylogeographic structure inferred above, the distribution of the *R. pusillus* species complex was divided into seven geographic regions (Figure 1). Bayesian phylogenetic trees generated in the previous analyses were used as input, and the maximum number of ancestral areas was limited to two. Default settings were used for S-DIVA. For BBM analyses, the F81+gamma substitution model was applied with ten MCMC chains, a temperature of 0.1, five million generations, and sampling every 100 generations. The first 25% of samples were discarded as burn-in.

To provide a broad-scale environmental context for the phylogeographic history of the focal mainland–island assemblage, a single pooled-occurrence ecological niche model was constructed using MaxEnt v3.4.0 [57]. The focal assemblage was operationally defined as nominal *R. pusillus* from mainland China and Hainan Island together with the Taiwan-endemic nominal species *R. monoceros*. Occurrence records were compiled from the present study and field surveys conducted since 2002, comprising 138 localities for *R. pusillus* and 21 localities for *R. monoceros*. These 159 occurrence records were pooled because the purpose of the modelling was to characterize broad temporal changes in potential habitat availability across the complete mainland–Hainan–Taiwan system, rather than to estimate separate ecological niches for the two nominal taxa. The inclusion of *R. monoceros* did not imply taxonomic synonymy or ecological equivalence between the two taxa, and the pooled model was not used to test niche equivalency, niche differentiation or species boundaries.

Climatic variables representing current conditions (1960–1990) and the Last Glacial Maximum (LGM; approximately 22 ka) were obtained from the WorldClim database [58]. Future climate projections for 2080–2099 were obtained from the Climate Change, Agriculture and Food Security database. Elevation, land-cover and vegetation data were obtained from the Global Map archives. All environmental layers were processed in QGIS Desktop v2.10.1. The candidate predictor set therefore included climatic, elevational, land-cover and vegetation variables. To reduce multicollinearity among predictors, Pearson correlation coefficients were calculated using environmental values extracted from 1000 randomly generated locations across the study area together with all occurrence localities. Correlation analyses were performed in IBM SPSS Statistics v23.0 and QGIS, and only variables with pairwise correlations below |r| = 0.9 were retained for model construction. The MaxEnt model was calibrated using 75% of the pooled occurrence records, with the remaining 25% withheld as test data. All other parameters were retained at their default settings. Habitat suitability maps for current, Last Glacial Maximum and future climatic conditions were generated in QGIS.

## 3. Results

### 3.1. Phenotypic Variation

Acoustic and morphological analyses revealed substantial geographic variation across the *R. pusillus* species complex, although the magnitude of differentiation differed among traits. A total of 372 *R. pusillus* and 554 *R. monoceros* call-frequency records were analyzed (Figure S1a). Females consistently emitted higher resting frequencies (RFs) than males throughout the complex. Among *R. pusillus* populations, South China (SC) showed the greatest acoustic variation and broad overlap with neighbouring geographic groups. Within SC, individuals from the central region emitted calls approximately 10 kHz lower than those from the southernmost and northwestern populations. In contrast, RF varied comparatively little among most other geographic groups, except for Southwest China (SWC), which consistently exhibited the lowest resting frequencies.

Morphological variation showed a similar but less pronounced geographic pattern (Figure S1b). Individuals from YM had relatively larger forearm lengths and greater body mass than those from other regions, whereas Hainan (HN) populations had comparatively lower body mass. SC populations overlapped extensively with neighbouring populations in both forearm length and body mass. Significant geographic variation was also detected in forearm mass index (FMI), used here as a proxy for relative wing loading (Figure S1c; one-way ANOVA, F_5_ = 15.49, *p* < 0.001). YM populations exhibited significantly higher FMI values than other mainland populations, whereas HN populations showed the lowest values. Overall, acoustic traits, body size, and FMI consistently indicated that SC populations occupy an intermediate phenotypic position linking multiple regional groups, while YM and HN populations showed the most distinctive phenotypic characteristics within *R. pusillus*.

### 3.2. Mitochondrial Diversity and Phylogeographic Structure

The mitochondrial datasets revealed high genetic diversity across the *R. pusillus* species complex. A total of 278 newly generated and 16 previously published *Cyt b* sequences produced a 1070 bp alignment containing 148 haplotypes and 160 polymorphic sites. The D-loop dataset comprised 253 newly generated and 89 published sequences, yielding 250 haplotypes across a 391 bp alignment with 138 polymorphic sites. For ND1, 123 newly generated sequences generated 48 haplotypes from a 957 bp alignment containing 68 polymorphic sites. Sampling localities and GenBank accession numbers are provided in Table S3.

**Figure 2:**
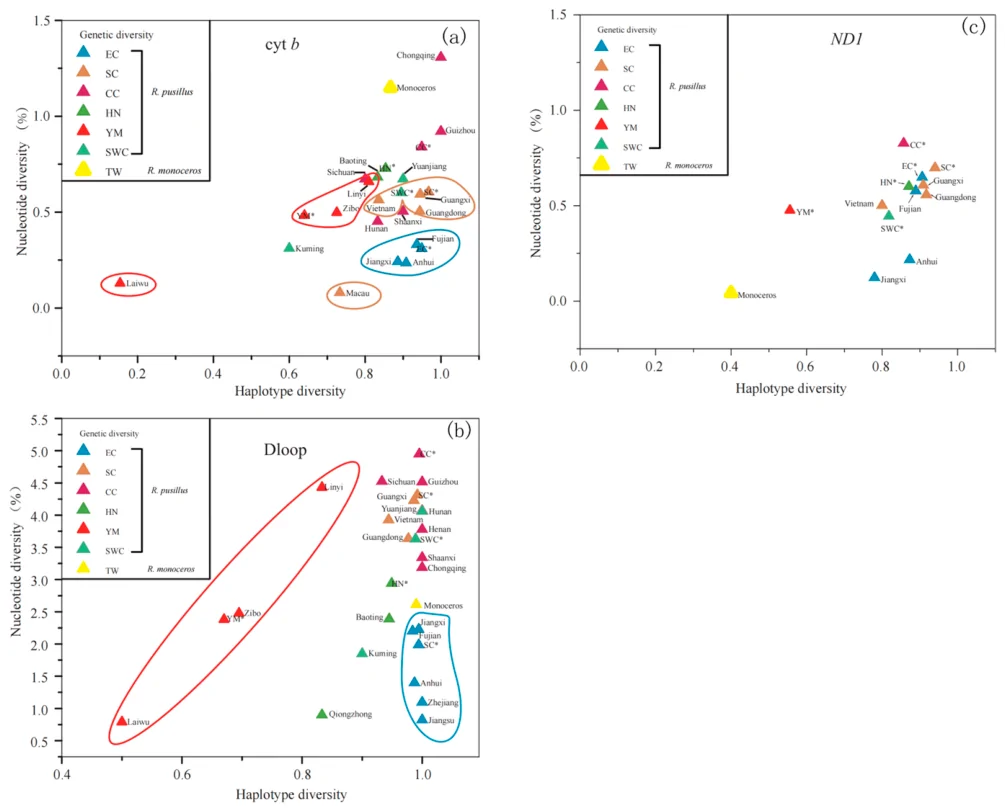
Mitochondrial genetic diversity of *R. pusillus* geographic groups and *R. monoceros*. Relationships between haplotype diversity and nucleotide diversity are shown for three mitochondrial markers: (**a**) *Cyt b*, (**b**) D-loop and (**c**) ND1. Symbols represent geographic groups of *R. pusillus* and the Taiwan lineage of *R. monoceros*, with group abbreviations and colours corresponding to Figure 1. Coloured ellipses visually highlight populations showing similar genetic-diversity patterns within selected geographic groups; blue, orange and red ellipses correspond to East China, South China and the Yimeng Mountains, respectively. Asterisks indicate genetic diversity estimates calculated from pooled samples within each geographic group, whereas locality names represent estimates for individual sampling populations.

Mitochondrial diversity varied markedly among geographic groups (Table S5). SC exhibited the highest haplotype diversity, whereas East China (EC) maintained high haplotype diversity but comparatively low nucleotide diversity. In contrast, YM consistently showed the lowest mitochondrial diversity across all three markers, particularly in haplotype diversity. Geographic variation in mitochondrial diversity broadly paralleled the phenotypic patterns described above (Figure 2; Table S5), with SC showing extensive overlap with neighbouring groups and EC showing comparatively greater genetic distinctiveness. The relatively high ND1 nucleotide diversity observed in Fujian populations within EC probably reflects limited sampling for this marker rather than a genuine increase in genetic variation.

Bayesian inference (BI) and maximum-likelihood (ML) analyses of *Cyt b* recovered highly congruent topologies and consistently supported the monophyly of the *R. pusillus* species complex (Figure 1 and Figure 2). Within *R. pusillus*, five geographically structured mitochondrial lineages were identified, corresponding to East China (EAST), South China (SOUTH), Central China (CENTER), Hainan Island (HN), and Southwest China (Yunnan; SWC). Four lineages received strong support (Bayesian posterior probability > 0.90; ML bootstrap support > 91%), whereas the CENTER lineage was weakly resolved (BPP < 0.50; BS < 70%). Although each lineage had a distinct geographic centre, haplotypes were frequently shared among neighbouring regions, especially around SC. Haplotypes H15, H19 and H22 occurred in both SC and EC, whereas H40 was shared between SC and HN. Long-distance haplotype sharing was uncommon, except for H6, which occurred in both EC and the geographically distant YM populations.

D-loop phylogenies showed weaker support for deep relationships among mainland lineages, but the Yunnan lineage and *R. monoceros* remained strongly resolved (BPP = 1.00), with positions congruent with the *Cyt b* results. The YM population showed an unusual pattern, with individuals carrying haplotypes belonging to nearly all mainland mitochondrial lineages except the Yunnan lineage, suggesting a complex evolutionary history rather than independent long-term isolation.

**Figure 3:**
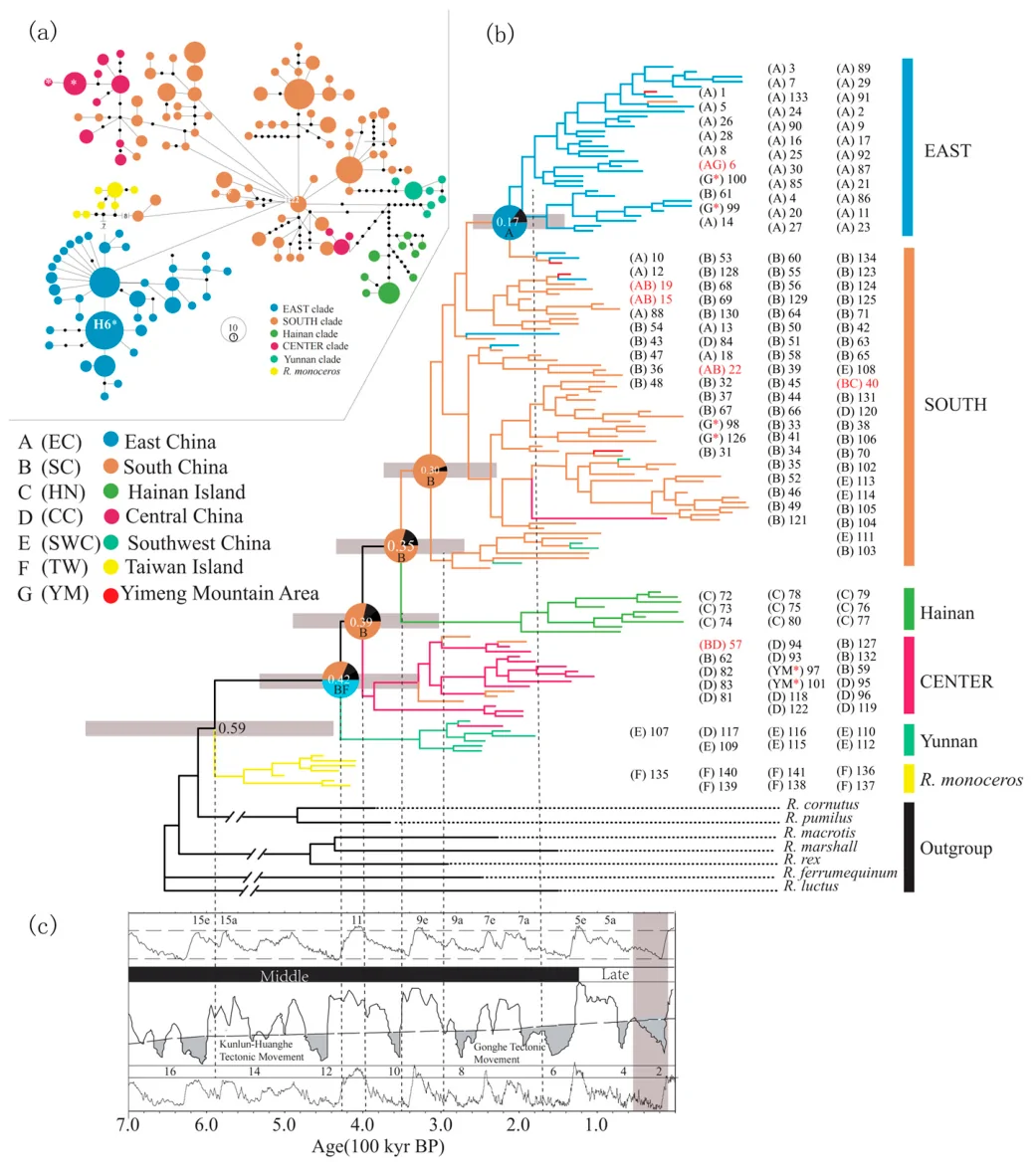
*Cyt b* phylogeny (**a**), haplotype network (**b**), divergence times and ancestral-area reconstruction (**c**) for *R. pusillus* and *R. monoceros*. The Bayesian phylogeny was reconstructed from *Cyt b* sequences and shows the major mitochondrial lineages within *R. pusillus* and the Taiwan lineage of *R. monoceros*. Lineage colours correspond to those used in Figure 1. Geographic regions associated with each haplotype are indicated by letters: A, EC; B, SC; C, HN; D, CC; E, SWC; F, TW; and G, YM. Haplotypes occurring in more than one geographic region are highlighted in red, and asterisks indicate haplotypes sampled from YM. Ancestral areas were inferred using S-DIVA based on the seven geographic regions defined in Figure 1; pie charts at nodes indicate reconstructed ancestral-area probabilities, and the letters mark the most probable ancestral region. Numbers at nodes indicate mean divergence times in Ma, and grey bars represent 95% HPD intervals. Marine isotope stages and corresponding temperature changes are shown below the tree for temporal reference. The *Cyt b* haplotype network summarizes genealogical relationships among haplotypes; circle size is proportional to haplotype frequency, and black dots or numbers indicate mutational steps.

Haplotype network analyses based on *Cyt b* closely mirrored the phylogenetic results (Figure 3a). The network showed an overall star-like topology centred on the widespread ancestral haplotype H22, primarily distributed in SC but also present in EC. The EAST and CENTER lineages were closely connected to the ancestral SOUTH lineage by one to three mutational steps, whereas the HN and Yunnan lineages were separated from SOUTH by seven to nine mutations. The EAST lineage displayed a typical star-like expansion pattern centred on H6 and H16, while the SOUTH lineage showed a more reticulate structure with numerous low-frequency peripheral haplotypes. The CENTER lineage was divided into two subgroups, consistent with its weak phylogenetic support. In YM, haplotypes were mostly terminal within several mainland lineages, further indicating multiple mitochondrial origins rather than a distinct mitochondrial lineage.

Both pairwise ΦST estimates and AMOVA supported pronounced mitochondrial differentiation across the complex. Based on *Cyt b*, overall differentiation was high within *R. pusillus* (*ΦST* = 0.61, *p* < 0.001) and remained high when *R. monoceros* was included (*ΦST* = 0.64, *p* < 0.001). All pairwise comparisons among the major geographic lineages were significant (*p* < 0.001). D-loop analyses recovered lower global differentiation (*ΦST* = 0.31–0.32), indicating weaker phylogeographic resolution for this marker. Hierarchical AMOVA showed that 41.96–50.88% of the total mitochondrial variation was explained by differences among geographic groups, whereas 35.41–42.16% occurred within populations (Table 1). The strongest differentiation was detected between YM and CC, SC and HN, and SC and SWC, whereas differentiation between SC and EC was weaker.

Biologically, these estimates indicate that a substantial proportion of maternal genetic variation is spatially partitioned among regional groups rather than being distributed randomly across the species range. This pattern is consistent with the long-term regional persistence of mitochondrial lineages and restricted female-mediated connectivity over evolutionary timescales. However, the comparatively weak differentiation between SC and EC, together with their shared haplotypes, indicates recurrent or relatively recent maternal connectivity between neighbouring mainland regions. In contrast, the stronger differentiation involving HN, SWC and YM suggests more persistent geographic or demographic isolation. Thus, the high global *ΦST* values reflect pronounced but heterogeneous mitochondrial subdivision rather than complete isolation among all regional populations.

### 3.3. Divergence Time and Demographic History

Bayesian molecular dating based on *Cyt b* provided well-supported divergence estimates across the *R. pusillus* species complex, with all parameters showing excellent convergence (ESS > 800). The most recent common ancestor (TMRCA) of the clade containing *R. pusillus* and *R. monoceros* was estimated at 0.59 Ma (95% HPD: 0.44–0.75 Ma), whereas the crown age of *R. pusillus* was estimated at 0.42 Ma (95% HPD: 0.32–0.53 Ma). Divergence among major mainland mitochondrial lineages occurred during the Middle Pleistocene, with estimated ages ranging from approximately 0.13 to 0.35 Ma (Figure 3c), indicating diversification over a relatively short evolutionary interval.

Bayesian Skyline Plot analyses revealed dynamic demographic histories for both the entire complex and individual mitochondrial lineages (Figure S2). At the species-complex level, effective population size remained relatively stable during the early Middle Pleistocene, increased gradually from approximately 0.41 Ma, and showed accelerated growth after 0.25 Ma. Population expansion continued through much of the Late Pleistocene but became increasingly variable after approximately 0.07 Ma. Within *R. pusillus*, demographic trajectories were broadly similar, although the EAST and SOUTH lineages showed particularly rapid Late Pleistocene expansion, whereas the CENTER, Hainan and Yunnan lineages exhibited more gradual growth.

Mismatch distribution analyses further supported recent demographic expansion. Unimodal distributions were recovered for the entire complex, *R. pusillus*, and the EAST and SOUTH mitochondrial lineages (Figure S2), and the sudden expansion model could not be rejected in these cases (SSD, *p* > 0.05). Significantly negative Tajima’s D and Fu’s FS values, together with significant R_2_ statistics (Table S6), also indicated departures from demographic equilibrium consistent with recent expansion. Estimated expansion times were approximately 0.089 Ma for *R. pusillus*, 0.086 Ma for the entire complex, 0.083 Ma for the SOUTH lineage, and 0.027 Ma for the EAST lineage. These results indicate that lineage diversification occurred mainly during the Middle Pleistocene, followed by repeated demographic expansion during the Late Pleistocene.

### 3.4. Nuclear Sequence Variation and Microsatellite Population Structure

**Figure 4:**
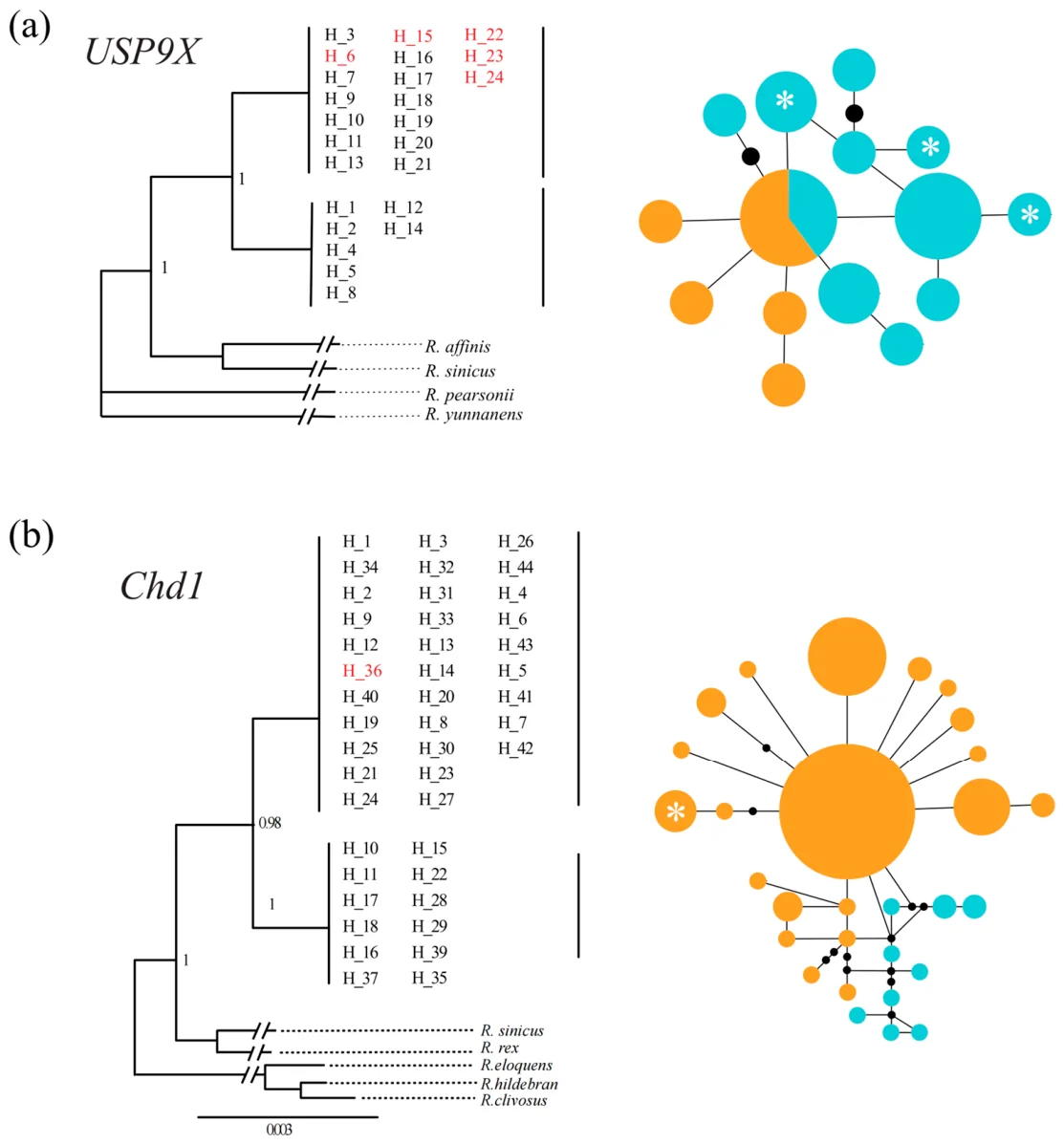
Nuclear genealogical relationships based on USP9x and Chd1. Bayesian phylogenetic trees and haplotype networks are shown for two nuclear loci: (**a**) USP9x and (**b**) Chd1. Haplotypes of *R. monoceros* are highlighted in red in the Bayesian trees and marked with asterisks in the haplotype networks. The networks illustrate the shallow nuclear differentiation and allele sharing between *R. monoceros* and mainland *R. pusillus*. In the networks, the asterisks represent the haplotypes from *Rhinolophus Monoceros* and black dots represents the mutational steps.

Nuclear sequence data showed much weaker geographic structure than the mitochondrial markers (Figure 4). Across the four nuclear loci examined, phylogenetic trees and haplotype networks did not recover the five mitochondrial lineages as reciprocally monophyletic groups. Instead, most mainland populations shared closely related or identical nuclear haplotypes, and genealogical relationships were generally shallow. The nuclear markers therefore provided limited support for deep subdivision among the East China, South China, Central China, Hainan and Southwest China mitochondrial lineages.

Among the nuclear loci, USP9x and Chd1 contained the clearest phylogeographic signal and were therefore most informative for visualizing genealogical relationships (Figure 4). In both loci, haplotypes from *R. pusillus* and *R. monoceros* were closely related, but *R. monoceros* tended to occupy relatively distinctive positions within the networks. However, allele sharing or close haplotype connections between Taiwan and mainland samples were still observed, indicating that nuclear differentiation between *R. monoceros* and mainland *R. pusillus* was incomplete. SWS1 and THY showed comparatively lower resolution and did not provide strong geographic separation among the major mainland groups.

Microsatellite analyses also revealed weaker population structure than mitochondrial DNA. STRUCTURE analyses across K = 2–6 indicated extensive admixture among most mainland populations, particularly among East China, South China and Central China (Figure S3). No sharp nuclear boundary corresponded to the five mitochondrial lineages recovered from *Cyt b*. Nevertheless, some regional differentiation was detected. YM showed the most distinctive microsatellite assignment among mainland populations, consistent with its phenotypic distinctiveness and reduced mitochondrial diversity. The Taiwan population corresponding to *R. monoceros* also showed partial nuclear differentiation from mainland *R. pusillus*, but this differentiation was weaker than the mitochondrial separation.

Microsatellite quality-control analyses indicated that the dataset was generally reliable. No significant linkage disequilibrium was detected among loci, and GenAlEx revealed no evidence of scoring artefacts or rare-allele excess. Departures from Hardy–Weinberg equilibrium were detected in EC, SC and YM after Bonferroni correction, mainly reflecting heterozygote deficiencies. However, BOTTLENECK detected no evidence of recent reductions in effective population size under the stepwise mutation model. LOSITAN identified PH57 and PH78 as potential outlier loci at the 95% confidence level, and these loci were excluded from subsequent analyses where appropriate. These results suggest that the observed departures from equilibrium most likely reflect historical population subdivision, local demographic effects and locus-specific variation rather than genotyping errors or recent demographic contraction.

Taken together, the nuclear sequence and microsatellite datasets revealed substantial mitonuclear discordance. Mitochondrial markers recovered strong geographic subdivision, whereas nuclear markers showed extensive allele sharing and comparatively weak differentiation among most mainland populations. This pattern is consistent with faster mitochondrial lineage sorting and more restricted female-mediated gene flow, whereas the widespread sharing of nuclear variation may reflect the prolonged retention of ancestral polymorphism and intermittent historical connectivity, including male-mediated gene flow. However, the relative contributions of these processes cannot be distinguished using the present marker set alone.

### 3.5. Historical Biogeography and Ecological Niche Modelling

Ancestral area reconstructions using both S-DIVA and Bayesian Binary MCMC (BBM) recovered broadly congruent biogeographic histories for the *R. pusillus* species complex (Figure 3b). Across the Bayesian phylogeny, both methods inferred 23 dispersal and 23 vicariance events among 40 reconstructed nodes, with high-confidence ancestral distributions recovered for 32 nodes (marginal probability > 0.60). Although the two approaches showed some disagreement at the deepest nodes, ancestral reconstructions within *R. pusillus* were highly consistent.

The earliest divergence within the *R. pusillus* species complex was reconstructed from an ancestral distribution spanning South China–Taiwan (BF), followed by dispersal into Southwest China and a vicariance event separating *R. monoceros* from *R. pusillus* (Node 1; Figure 3b). Within *R. pusillus*, Nodes 2–4 consistently supported South China as the principal ancestral area, followed by repeated dispersal into East China and subsequent geographic isolation among regional lineages. The Hainan lineage showed a more complex history involving multiple dispersal events between mainland Southeast Asia, Central China and Hainan Island before its present distribution was established (Nodes 5–7). For these nodes, S-DIVA and BBM were highly concordant, with most marginal probabilities exceeding 0.70. BBM inferred more dispersal events than S-DIVA, particularly among adjacent regions, but the inferred sequence of major lineage divergence remained largely unchanged. Both methods therefore supported repeated cycles of dispersal followed by geographic isolation during the evolutionary history of the complex.

**Figure 5:**
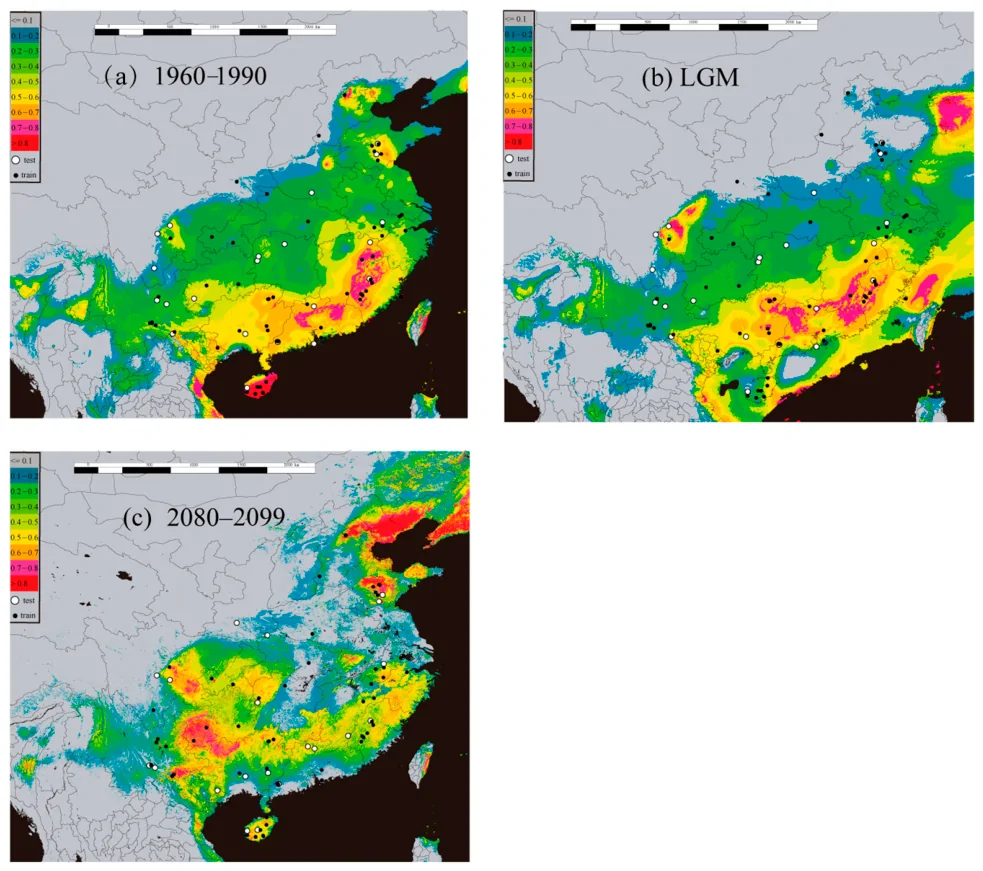
Pooled-occurrence ecological niche model for the focal *Rhinolophus pusillus–R. monoceros* mainland–island assemblage under different climatic periods. Predicted environmental suitability based on the pooled occurrence records of nominal *R. pusillus* from mainland China and Hainan Island and the Taiwan-endemic nominal species *R. monoceros* is shown for (**a**) current climatic conditions, (**b**) the Last Glacial Maximum (LGM), and (**c**) future climatic conditions for 2080–2099. Habitat suitability is represented by a colour gradient from low to high suitability, with warmer colours indicating higher predicted suitability. The maps represent broad-scale potential habitat availability across the focal assemblage and should not be interpreted as independent species-specific niche models.

The pooled-occurrence ecological niche model predicted broad temporal changes in potential environmental suitability across the East Asian mainland–island system (Figure 5). Under current climatic conditions, the highest predicted suitability was concentrated in the mountainous regions of South China, particularly around the Nanling and Wuyi Mountains, with an additional area of relatively high suitability in the Yimeng Mountains. During the Last Glacial Maximum, potentially suitable habitat extended broadly across mainland East and South China but showed greater regional fragmentation, whereas predicted suitability was reduced on Hainan, Taiwan and in the Yimeng Mountains. Two additional areas of relatively high suitability were predicted near the Taiwan Strait and north of the Yimeng Mountains. Future projections indicated a northward shift and increasing fragmentation of potentially suitable habitat, with increased suitability across parts of the Yungui Plateau and northern regions and reduced suitability in parts of Hainan and Taiwan.

Because the occurrence records of *R. pusillus* and *R. monoceros* were pooled, these maps represent broad-scale temporal changes in potential habitat availability across the focal mainland–island assemblage. They should not be interpreted as independent species-specific predictions or as evidence that the two nominal taxa occupy equivalent ecological niches. Together with the ancestral-area reconstruction, the model provides a broad environmental context for a biogeographic history involving repeated changes in habitat suitability, dispersal opportunities and geographic isolation.

## 4. Discussion

The present study provides a range-wide phylogeographic framework for the *Rhinolophus pusillus* species complex across the East Asian mainland–island system. By integrating mitochondrial and nuclear sequence data, microsatellite genotypes, phenotypic traits, demographic inference, ancestral-area reconstruction, and ecological niche modelling, we show that diversification within this complex is geographically structured but evolutionarily dynamic. Mitochondrial DNA revealed pronounced regional subdivision, whereas nuclear loci and microsatellites indicated extensive allele sharing and weaker geographic structure. This contrast suggests that the complex has not diversified through long-term complete isolation alone, but through repeated cycles of regional persistence, dispersal, isolation, and secondary contact.

Mitochondrial analyses identified five principal lineages within *R. pusillus*, corresponding broadly to South China, East China, Central China, Hainan Island, and Southwest China, while *R. monoceros* formed a closely related but distinctive Taiwan lineage. These lineages showed clear geographic centres and significant mitochondrial differentiation, indicating that historical isolation has contributed substantially to regional divergence. Among them, South China occupied a particularly important position. It showed the highest mitochondrial diversity, contained or was closely connected to several widespread haplotypes, and was repeatedly inferred as an ancestral area in biogeographic reconstruction. This pattern suggests that South China served both as a long-term refugial region and as a source area for subsequent dispersal.

The importance of South China is consistent with broader phylogeographic patterns in East Asia. Unlike regions strongly affected by continental ice sheets, East Asia experienced spatially heterogeneous Quaternary climatic change, allowing many organisms to persist in multiple regional refugia [12,13,53,59]. The complex topography, karst systems, river valleys, and forested mountains of southern China may have provided stable habitats for cave-roosting and forest-associated bats while also promoting local differentiation. In *R. pusillus*, the high diversity and central genealogical position of South China populations probably reflect long-term persistence, retention of ancestral variation, and repeated contact with neighbouring regions.

In contrast, the Southwest China lineage appears to represent a more deeply isolated evolutionary unit. Its early divergence, mitochondrial distinctiveness, and separate demographic trajectory suggest prolonged regional isolation. Southwestern China is widely recognized as a centre of biodiversity and endemism, shaped by complex topography and elevational gradients. Although our data do not directly test the effects of specific geological events, the distinctiveness of the Yunnan lineage is compatible with the role of this region as a semi-isolated refugium. Populations there may have persisted independently through multiple climatic cycles before limited later contact with other mainland lineages.

The East China and Central China lineages showed closer relationships with South China. Haplotype networks placed these lineages near South China haplotypes, and several haplotypes were shared among neighbouring regions. This suggests that diversification in the eastern and central mainland was not caused by a single episode of isolation, but by repeated expansion, local differentiation, and secondary contact. The East China lineage, in particular, showed a star-like network and demographic evidence of Late Pleistocene expansion, indicating rapid population growth after earlier restriction or instability.

Comparable phylogeographic patterns have been reported in other East Asian bats. In southern China, Pleistocene climatic cycling promoted regional diversification and subsequent demographic changes in *Rhinolophus affinis*, whereas *Hipposideros armiger* retained signatures of persistence in multiple Pleistocene refugia across the Oriental Region [51,55]. In *H. larvatus*, differentiation between mainland China and Hainan Island was shaped jointly by colonization history, geographic separation and environmental variation [60]. Together, these studies suggest that southern China and its adjacent continental-shelf islands did not function as a single continuous refugium, but rather as a mosaic of persistent regional populations whose connectivity changed repeatedly through time. The *R. pusillus* complex conforms broadly to this regional pattern, although the exceptionally high mitochondrial diversity and central genealogical position of the South China populations identify this region particularly clearly as both a centre of persistence and a source of subsequent dispersal. At the same time, the comparatively weak nuclear subdivision indicates that historical connectivity among mainland populations remained substantial.

The island lineages further illustrate the dynamic nature of diversification in this complex. Hainan has been repeatedly connected to and isolated from the mainland during Quaternary sea-level fluctuations. The mitochondrial distinctiveness of the Hainan lineage indicates that isolation was sufficient to promote divergence, whereas haplotype sharing and ancestral-area reconstruction suggest that its history also involved intermittent mainland connection. Thus, Hainan does not appear to represent either a simple mainland extension or a lineage shaped by permanent isolation. Instead, its evolutionary history likely reflects alternating dispersal and vicariance.

Taiwan and *R. monoceros* represent another important case of insular divergence. Previous studies differed in whether *R. monoceros* should be treated as a distinct species or as an insular form closely related to *R. pusillus* [8,10]. Our results support both close affinity and evolutionary distinctiveness. *R. monoceros* is closely related to mainland *R. pusillus*, but it forms a geographically distinctive Taiwan lineage and has previously been shown to differ morphologically. Repeated Pleistocene land-bridge formation may have allowed historical exchange between Taiwan and the mainland, whereas subsequent sea-level rise restored isolation and promoted independent differentiation. The close relationship between the two forms therefore does not contradict the distinctiveness of *R. monoceros*, but instead reflects a recent and dynamic mainland–island history.

Similar processes have shaped the phylogeographic histories of non-volant mammals in the region, indicating that the pattern observed here is not restricted to bats. The rice field mouse *Mus caroli* contains mitochondrial lineages that probably originated during the Middle Pleistocene, including a Taiwan-restricted lineage associated with regional palaeogeographic and sea-level changes; in contrast, differentiation among mainland lineages appears to have involved dispersal across geographic barriers followed by local differentiation [61]. Père David’s vole, *Eothenomys melanogaster*, also exhibits Pleistocene divergence among southeastern, southwestern and central montane regions of South China, while climatic cycles promoted alternating isolation, population admixture and episodes of connectivity between the mainland and Taiwan [62]. These comparisons support a broader East Asian biogeographic model in which topographic heterogeneity and climatic instability preserve geographically structured lineages, whereas lowered sea levels and climatically driven range shifts periodically restore connectivity. The *R. pusillus* complex extends this pattern to a highly mobile mammal, demonstrating that flight may facilitate secondary contact without completely erasing the genetic signatures of regional refugia and island isolation.

Divergence-time estimates further support the importance of Quaternary environmental change. The origin of the *R. pusillus* species complex was dated to the Middle Pleistocene, and diversification among major mainland lineages occurred over a relatively short interval. This period was characterized by repeated climatic oscillations, vegetation shifts, and sea-level changes across East Asia. These processes likely caused recurrent changes in habitat suitability and connectivity, promoting regional isolation during unfavourable periods and expansion or secondary contact during favourable intervals. Such a scenario explains the coexistence of strong mitochondrial structure, shared haplotypes among neighbouring populations, and demographic expansion in several lineages.

Demographic analyses showed that expansion occurred mainly during the Late Pleistocene. Bayesian skyline plots, mismatch distributions, and neutrality tests supported population growth in the complex as a whole and especially in the South China and East China lineages. The timing of expansion indicates that regional lineages first diverged during the Middle Pleistocene and later expanded from refugial or semi-refugial areas. However, expansion was not uniform. Hainan and Yunnan showed more restricted or gradual demographic changes, suggesting that local geography, habitat continuity, and island isolation strongly influenced demographic responses to climatic change.

Ecological niche modelling was used here to provide a broad-scale environmental context for the phylogeographic and demographic history of the focal mainland–island assemblage. The predicted persistence of extensive suitable habitat across South China during the Last Glacial Maximum is geographically consistent with the exceptionally high mitochondrial diversity, the central positions of widespread haplotypes and the ancestral-area reconstructions identifying this region as an important area of persistence and dispersal. Similarly, the broad distribution of potentially suitable habitat across parts of South and East China is compatible with the close genealogical relationships and haplotype sharing between the SOUTH and EAST lineages. Together with the star-like EAST haplotype network and its inferred Late Pleistocene demographic expansion, these patterns are consistent with expansion from southern or southeastern mainland refugial areas followed by recurrent contact between neighbouring regions.

In contrast, the predicted reduction and fragmentation of suitable habitat on Hainan and Taiwan during the Last Glacial Maximum are geographically consistent with the restricted distributions and pronounced mitochondrial distinctiveness of the Hainan lineage and *R. monoceros*. These island populations may have experienced stronger periods of geographic isolation than the more continuously connected South and East China populations, although episodic land-bridge formation could have permitted occasional dispersal. These spatial correspondences support a general historical scenario involving mainland persistence, repeated range expansion and secondary contact, together with stronger isolation in peripheral island regions. They should nevertheless be regarded as geographic consistency between independent lines of evidence rather than as direct demonstrations of particular refugia, dispersal routes or causal effects of climate.

An important limitation is that occurrence records of *R. pusillus* and *R. monoceros* were pooled in the ecological niche model. The resulting predictions therefore represent the broad potential environmental envelope of the focal assemblage rather than the realized or fundamental niche of either nominal taxon. The model cannot determine whether *R. monoceros* occupies an ecological niche distinct from that of *R. pusillus*, nor can it provide taxon-specific estimates of past refugial distribution, future range change or climatic vulnerability. Accordingly, the predicted changes on Hainan and Taiwan are interpreted here as regional environmental hypotheses that are consistent with stronger island isolation, rather than as direct evidence of species-specific range contraction. Separate taxon-specific models with adequate occurrence records, together with formal niche-equivalency and niche-similarity tests, would be required to evaluate ecological differentiation between *R. pusillus* and *R. monoceros*.

Mitonuclear discordance is central to interpreting the evolutionary history of this complex. Mitochondrial DNA showed strong geographic subdivision, whereas nuclear loci and microsatellites revealed much weaker structure and widespread allele sharing. Part of this geographic differentiation may reflect isolation by distance, because gene flow is generally expected to decline as geographic distance increases. Female natal philopatry combined with greater male-mediated dispersal may further maintain strong mitochondrial structure while reducing differentiation in nuclear markers. Together with incomplete lineage sorting and historical connectivity, these processes may have contributed to the observed mitonuclear discordance. Similar contrasts between geographically structured mitochondrial lineages and weakly differentiated nuclear populations have been repeatedly documented in bats, including *Myotis myotis*, *R. ferrumequinum*, *R. affinis* and *R. monoceros* [22,28,51,63]. One important explanation is female natal philopatry combined with greater male-mediated dispersal. Under this scenario, maternally inherited mitochondrial haplotypes remain geographically localized because females return to or remain near their natal breeding areas, whereas dispersing males transfer nuclear alleles among populations and reduce nuclear differentiation. This mechanism is particularly relevant to the present complex because previous analyses of *R. monoceros* found substantially stronger population structure in mitochondrial than in autosomal markers, consistent with female-biassed philopatry and male-biassed dispersal.

Differences in the evolutionary properties of the markers probably contributed as well. Because mitochondrial DNA is haploid, maternally inherited and has a smaller effective population size than most nuclear loci, mitochondrial lineages are more strongly affected by genetic drift and generally reach reciprocal monophyly more rapidly. In contrast, nuclear loci can retain ancestral polymorphisms for considerably longer periods following population divergence. Given the relatively recent Middle Pleistocene diversification inferred here, incomplete lineage sorting is therefore expected to remain prominent in the nuclear genome. Intermittent historical contact or introgression during periods of increased habitat connectivity could have further homogenized nuclear variation, particularly among the South, East and Central China populations. Our data do not directly estimate sex-specific dispersal or distinguish quantitatively among incomplete lineage sorting, sex-biassed gene flow and historical introgression. The observed discordance is therefore best interpreted as the probable combined outcome of these processes rather than as evidence for any single mechanism.

This discordance has important taxonomic implications. If mitochondrial data were considered alone, the five *R. pusillus* lineages might appear to represent strongly differentiated evolutionary units. Conversely, nuclear data alone would suggest broad continuity among mainland populations. Neither view fully captures the evolutionary history of the complex. Instead, the evidence indicates a continuum of divergence. Yunnan, Hainan, and Taiwan show stronger historical isolation, whereas South, East, and Central China retain clearer evidence of recurrent connectivity. Such a pattern is typical of young species complexes in which genetic, phenotypic, and ecological differentiation accumulate at different rates [63,64,65,66].

Within this framework, a conservative taxonomic interpretation is appropriate. The combined genetic, morphological and acoustic evidence supports the recognition of *R. monoceros* as a distinct species closely related to *R. pusillus*. In contrast, the major mainland and Hainan lineages are best regarded as important phylogeographic units pending further integrative taxonomic assessment, rather than being immediately elevated to formal taxonomic status. In particular, the Hainan population, historically referred to as *R. p. parcus*, shows mitochondrial distinctiveness and phenotypic differences, but also evidence of historical mainland connection. These results support its recognition as a significant regional lineage, while indicating that formal taxonomic revision should await genome-wide evidence, reproductive data, and more detailed ecological comparisons.

Phenotypic variation further supports regional differentiation but does not map perfectly onto genetic structure. Resting frequency, forearm length, body mass, and FMI varied geographically, with YM and Hainan showing the most distinctive patterns, whereas South China overlapped broadly with neighbouring regions. In horseshoe bats, echolocation and morphology may be influenced by both evolutionary history and local ecological adaptation. The phenotypic differences observed here may therefore reflect adaptation to local habitat structure, prey availability, or foraging conditions, as well as drift. Because phenotypic patterns were not fully congruent with mitochondrial or nuclear structure, they are best treated as complementary evidence rather than diagnostic criteria for species delimitation.

From a conservation perspective, our results show that evolutionary diversity within this widespread complex is unevenly distributed. The Yunnan lineage represents an early divergent mainland lineage, Hainan reflects an insular population shaped by repeated isolation and reconnection, and Taiwan corresponds to the endemic *R. monoceros* lineage. These regional populations preserve distinct components of the evolutionary history of the complex. The YM population also deserves attention because of its low genetic diversity, distinct microsatellite assignment, and unusual mixed mitochondrial ancestry. Conservation planning should therefore consider major phylogeographic lineages and distinctive marginal populations, even where formal taxonomic boundaries remain unresolved.

Overall, the *R. pusillus* species complex represents an ongoing diversification system rather than a set of sharply separated evolutionary units. Its history was shaped by Quaternary climatic oscillations, regional topographic complexity, fluctuating sea levels, and repeated changes in habitat connectivity. South China appears to have served as a major centre of persistence and dispersal, Southwest China preserved an early divergent lineage, Hainan and Taiwan accumulated insular differentiation under intermittent mainland connectivity, and northern peripheral populations retained evidence of complex colonization. These findings reconcile earlier conflicting interpretations and highlight the importance of integrative phylogeography for resolving evolutionary histories and conserving hidden diversity in widespread bat species complexes, particularly in *Rhinolophus*, where recent studies continue to reveal rapid diversification, cryptic diversity and complex histories of dispersal, isolation and secondary contact [4,67].

## 5. Conclusions

This study provides an integrative phylogeographic assessment of the *Rhinolophus pusillus* species complex across the East Asian mainland–island system. Mitochondrial data revealed five geographically structured lineages within *R. pusillus*, corresponding broadly to South China, East China, Central China, Hainan Island and Southwest China, while *R. monoceros* formed a closely related but distinctive Taiwan lineage. South China was identified as a major centre of genetic diversity, ancestral persistence and historical dispersal.

In contrast, nuclear loci and microsatellites showed weaker geographic structure and extensive allele sharing, indicating incomplete lineage sorting and historical connectivity among recently diverged populations. Divergence-time estimates and demographic analyses indicate that Quaternary climatic oscillations and sea-level changes contributed to repeated periods of isolation, expansion and secondary contact. The pooled-occurrence ecological niche model provided broad-scale environmental context that was geographically consistent with this interpretation, but it was not used to infer taxon-specific ecological niches, past distributions or future range responses. Overall, the *R. pusillus* complex represents an ongoing diversification system, with several regional populations, particularly those from Southwest China, Hainan, Taiwan and the Yimeng Mountains, preserving important components of hidden evolutionary diversity.

## Figures and Tables

**Table 1:** Analysis of molecular variance (AMOVA) among geographic groups corresponding to mitochondrial lineages based on *Cyt b* sequences.

Population Group	Among Groups (%)	Among Populations Within Groups (%)	Within Populations (%)
EC/SC/CC/HN/YM/SWC/TW	48.84	14.94	36.23
EC/SC/CC/HN/YM/SWC	44.05	16.37	39.58
EC/SC/CC/HN/YM	42.29	16.63	41.08
EC/SC/CC/HN	41.96	15.88	42.16
SC/HN	50.88	13.3	35.74
SC/SWC	49.19 *	15.40	35.41
SC/CC	39.86	19.72	40.42
SC/EC	29.83	18.19	51.99
YM/EC	16.34	13.20	70.46
YM/SC	31.84	21.45	46.72
YM/CC	57.33 *	26.54	16.13

* *p* < 0.05.

## Data Availability

DNA nucleotide sequences obtained in this study have been submitted to the NCBI Nucleotide database and assigned the accession numbers: USP9X (PZ860755–PZ860820), THY (PZ860539–PZ860754), SWS1 (PZ860453–PZ860538), CHD1 (PZ849643–PZ849768), D-loop (PZ817569–PZ817816), ND1 (PZ817451–PZ817568), and CYTB (PZ817178–PZ817450).

## Supplementary Materials

The following supporting information can be downloaded at: https://www.mdpi.com/article/10.3390/ani16172669/s1, Figure S1: Geographic variation in resting frequency and morphology of *R. pusillus* and *R. monoceros*. (**a**) Mean resting frequency (RF) of *R. pusillus* and *R. monoceros* from 40 populations arranged by latitude. Open symbols denote females and filled symbols denote males. Colours indicate geographic groups. (**b**) Forearm length (FA) and body mass of 167 *R. pusillus* and 536 *R. monoceros* individuals. (**c**) Forearm mass index (FMI) of 167 *R. pusillus* individuals, calculated as body mass divided by forearm length squared. FMI was used as a comparative index of relative body condition and flight-related morphology. Figure S2: Mismatch distributions and Bayesian skyline plots based on mitochondrial *Cyt b* sequences for the major lineages and taxonomic groups of the *Rhinolophus pusillus* species complex. (**a**–**h**) show mismatch distributions for (**a**) Yunnan, (**b**) CENTER, (**c**) Hainan, (**d**) SOUTH, (**e**) EAST, (**f**) *R. pusillus*, (**g**) *R. monoceros*, and (**h**) the combined *R. pusillus–R. monoceros* complex. Grey bars represent the observed distributions of pairwise nucleotide differences, and black lines represent the expected distributions under the sudden demographic expansion model. (**i**–**p**) show the corresponding Bayesian skyline plots for (**i**) Yunnan, (**j**) CENTER, (**k**) Hainan, (**l**) SOUTH, (**m**) EAST, (**n**) *R. pusillus*, (**o**) *R. monoceros*, and (**p**) the combined *R. pusillus–R. monoceros* complex. In the skyline plots, the black line represents the median estimate of effective population size through time, and the blue lines indicate the 95% highest posterior density intervals. Time is expressed in millions of years ago (Mya). Figure S3. STRUCTURE analysis of the *Rhinolophus pusillus* species complex based on microsatellite genotypes. Individual assignment probabilities are shown for K = 2–6. Each vertical bar represents one individual, and colours indicate the inferred proportions of ancestry from different genetic clusters. Individuals are grouped according to geographic population. Table S1: Primers used for PCR amplification and sequencing in this study. Table S2: Published sequences included in the phylogenetic analyses. GenBank sequences used for comparative phylogenetic analyses are listed by species, marker, accession number and sampling locality. Table S3: Haplotypes generated from *Cyt b* and D-loop sequences. Sampling localities, geographic groups, haplotype assignments and sequence information are provided for the mitochondrial *Cyt b* and D-loop datasets. Table S4: Mitochondrial genetic diversity of *R. pusillus* and selected bat species. Genetic diversity indices for *R. pusillus* are compared with published estimates from other bat species, with emphasis on *Rhinolophus* taxa and geographically isolated populations. Table S5: Mitochondrial genetic diversity of *R. pusillus* and *R. monoceros* geographic groups. Table S6: Demographic statistics for lineages showing evidence of historical expansion. Expansion time estimates, mismatch distribution statistics, neutrality-test results and R_2_ statistics are summarized for major mitochondrial lineages. N indicates the number of individuals; SSD indicates the sum of squared deviations; r indicates Harpending’s raggedness index.

## References

[1] Cheng R., Luo A.R., Orr M., Ge D.Y., Hou Z.E., Qu Y.H., Guo B.C., Zhang F., Sha Z.L., Zhao Z. (2025). Cryptic diversity begets challenges and opportunities in biodiversity research. Integr. Zool..

[2] Avise J.C. (2000). Phylogeography: The History and Formation of Species.

[3] Mammal Diversity Database (2026). Mammal Diversity Database.

[4] Guo W.J., Wu Y., He K., Hu Y.B., Wang X.Y., Yu W.H. (2023). Unraveling the macroevolution of horseshoe bats (Chiroptera: Rhinolophidae: *Rhinolophus*). Zool. Res..

[5] Dool S.E., Puechmaille S.J., Foley N.M., Allegrini B., Bastian A., Mutumi G.L., Maluleke T.G., Odendaal L.J., Teeling E.C., Jacobs D.S. (2016). Nuclear introns outperform mitochondrial DNA in inter-specific phylogenetic reconstruction: Lessons from horseshoe bats (Rhinolophidae: Chiroptera). Mol. Phylogenet. Evol..

[6] Francis C., Guillén A., Robinson M., Duckworth J.W., Salter R.E., Khounboline K. (1999). Order Chiroptera: Bats. Wildlife in Lao PDR: 1999 Status Report.

[7] Csorba G., Ujhelyi P., Thomas N. (2003). Horseshoe Bats of the World:(Chiroptera: Rhinolophidae).

[8] Li G., Jones G., Rossiter S.J., Chen S.-F., Parsons S., Zhang S. (2006). Phylogenetics of small horseshoe bats from East Asia based on mitochondrial DNA sequence variation. J. Mammal..

[9] Jiang T., Metzner W., You Y., Liu S., Lu G., Li S., Wang L., Feng J. (2010). Variation in the resting frequency of *Rhinolophus pusillus* in Mainland China: Effect of climate and implications for conservation. J. Acoust. Soc. Am..

[10] Wu Y., Motokawa M., Harada M., Thong V.D., Lin L.K., Li Y.C. (2012). Morphometric variation in the *pusillus* group of the genus *Rhinolophus* (Mammalia: Chiroptera: Rhinolophidae) in East Asia. Zool. Sci..

[11] Soisook P., Karapan S., Srikrachang M., Dejtaradol A., Nualcharoen K., Bumrungsri S., Oo S.S.L., Aung M.M., Bates P.J.J., Harutyunyan M. (2016). Hill Forest Dweller: A New Cryptic Species of *Rhinolophus* in the ‘*pusillus* Group’ (Chiroptera: Rhinolophidae) from Thailand and Lao PDR. Acta Chiropterologica.

[12] Hewitt G.M. (2000). The genetic legacy of the Quaternary ice ages. Nature.

[13] Hewitt G.M. (2004). Genetic consequences of climatic oscillations in the Quaternary. Philos. Trans. R. Soc. B.

[14] Koopman K. (1994). Chiroptera: Systematics. Handbook of Zoology.

[15] Zhang L., Jones G., Zhang J., Zhu G., Parsons S., Rossiter S.J., Zhang S. (2009). Recent Surveys of Bats (Mammalia: Chiroptera) from China. I. Rhinolophidae and Hipposideridae. Acta Chiropterologica.

[16] Chen S.F., Rossiter S.J., Faulkes C.G., Jones G. (2006). Population genetic structure and demographic history of the endemic Formosan lesser horseshoe bat (*Rhinolophus monoceros*). Mol. Ecol..

[17] Chen S.F., Jones G., Rossiter S.J. (2009). Determinants of echolocation call frequency variation in the Formosan lesser horseshoe bat (*Rhinolophus monoceros*). Proc. Biol. Sci..

[18] Shen Q., Liu Q., Chen Y., Zhao J., Peng X., Sun Y., Chen M., Zhang L. (2015). Geographical variation in echolocation calls of least horseshoe bat (*Rhinolophus pusillus*) in China. Chin. J. Zool..

[19] Meng F., Zhu L., Huang W., Irwin D.M., Zhang S. (2016). Bats: Body mass index, forearm mass index, blood glucose levels and SLC2A2 genes for diabetes. Sci. Rep..

[20] Cao Y., Janke A., Waddell P.J., Westerman M., Takenaka O., Murata S., Okada N., Pääbo S., Hasegawa M. (1998). Conflict among individual mitochondrial proteins in resolving the phylogeny of eutherian orders. J. Mol. Evol..

[21] Lim B.K., Engstrom M.D., Bickham J.W., Patton J.C. (2007). Molecular phylogeny of New World sheath-tailed bats (Emballonuridae: Diclidurini) based on loci from the four genetic transmission systems in mammals. Biol. J. Linn. Soc..

[22] Mao X., Zhang J., Zhang S., Rossiter S.J. (2010). Historical male-mediated introgression in horseshoe bats revealed by multilocus DNA sequence data. Mol. Ecol..

[23] Matthee C.A., Burzlaff J.D., Taylor J.F., Davis S.K. (2001). Mining the mammalian genome for artiodactyl systematics. Syst. Biol..

[24] Stephens M., Smith N.J., Donnelly P. (2001). A New Statistical Method for Haplotype Reconstruction from Population Data. Am. J. Hum. Genet..

[25] Thompson J.D., Gibson T.J., Plewniak F., Jeanmougin F., Higgins D.G. (1997). The CLUSTAL_X windows interface: Flexible strategies for multiple sequence alignment aided by quality analysis tools. Nucleic Acids Res..

[26] Tamura K., Stecher G., Peterson D., Filipski A., Kumar S. (2013). MEGA6: Molecular evolutionary genetics analysis version 6.0. Mol. Biol. Evol..

[27] Hua P., Guo T., Liu W., Zhang S., Rossiter S.J. (2008). Isolation and characterization of 13 microsatellite loci in *Rhinolophus pusillus* (least horseshoe bat) with cross-amplification in five related species. Conserv. Genet..

[28] Flanders J., Jones G., Benda P., Dietz C., Zhang S., Li G., Sharifi M., Rossiter S.J. (2009). Phylogeography of the greater horseshoe bat, *Rhinolophus ferrumequinum*: Contrasting results from mitochondrial and microsatellite data. Mol. Ecol..

[29] Dool S.E., Puechmaille S.J., Dietz C., Juste J., Ibanez C., Hulva P., Roue S.G., Petit E.J., Jones G., Russo D. (2013). Phylogeography and postglacial recolonization of Europe by *Rhinolophus hipposideros*: Evidence from multiple genetic markers. Mol. Ecol..

[30] Librado P., Rozas J. (2009). DnaSP v5: A software for comprehensive analysis of DNA polymorphism data. Bioinformatics.

[31] Ronquist F., Teslenko M., van der Mark P., Ayres D.L., Darling A., Hohna S., Larget B., Liu L., Suchard M.A., Huelsenbeck J.P. (2012). MrBayes 3.2: Efficient Bayesian phylogenetic inference and model choice across a large model space. Syst. Biol..

[32] Stamatakis A. (2014). RAxML version 8: A tool for phylogenetic analysis and post-analysis of large phylogenies. Bioinformatics.

[33] Posada D. (2008). jModelTest: Phylogenetic model averaging. Mol. Biol. Evol..

[34] Posada D., Crandall K.A. (2001). Intraspecific gene genealogies: Trees grafting into networks. Trends Ecol. Evol..

[35] Mao X., He G., Zhang J., Rossiter S.J., Zhang S. (2013). Lineage divergence and historical gene flow in the Chinese horseshoe bat (*Rhinolophus sinicus*). PLoS ONE.

[36] Templeton A.R., Crandall K.A., Sing C.F. (1992). A cladistic analysis of phenotypic associations with haplotypes inferred from restriction endonuclease mapping and DNA sequence data. III. Cladogram estimation. Genetics.

[37] Clement M., Posada D., Crandall K.A. (2000). TCS: A computer program to estimate gene genealogies. Mol. Ecol..

[38] Leigh J.W., Bryant D. (2015). POPART: Full-feature software for haplotype network construction. Methods Ecol. Evol..

[39] Pritchard J.K., Stephens M., Donnelly P. (2000). Inference of population structure using multilocus genotype data. Genetics.

[40] Jakobsson M., Rosenberg N.A. (2007). CLUMPP: A cluster matching and permutation program for dealing with label switching and multimodality in analysis of population structure. Bioinformatics.

[41] Rosenberg N.A. (2004). DISTRUCT: A program for the graphical display of population structure. Mol. Ecol. Resour..

[42] Earl D.A. (2012). STRUCTURE HARVESTER: A website and program for visualizing STRUCTURE output and implementing the Evanno method. Conserv. Genet. Resour..

[43] Peakall R., Smouse P.E. (2012). GenAlEx 6.5: Genetic analysis in Excel. Population genetic software for teaching and research--an update. Bioinformatics.

[44] Piry S., Luikart G., Cornuet J.M. (1999). BOTTLENECK: A computer program for detecting recent reductions in the effective population size using allele frequency data. J. Hered..

[45] Antao T., Lopes A., Lopes R.J., Beja-Pereira A., Luikart G. (2008). LOSITAN: A workbench to detect molecular adaptation based on a F ST-outlier method. BMC Bioinform..

[46] Excoffier L., Lischer H.E. (2010). Arlequin suite ver 3.5: A new series of programs to perform population genetics analyses under Linux and Windows. Mol. Ecol. Resour..

[47] Drummond A.J., Rambaut A. (2007). BEAST: Bayesian evolutionary analysis by sampling trees. BMC Evol. Biol..

[48] Sanchez-Gracia A., Castresana J. (2012). Impact of deep coalescence on the reliability of species tree inference from different types of DNA markers in mammals. PLoS ONE.

[49] Nesi N., Kadjo B., Pourrut X., Leroy E., Pongombo Shongo C., Cruaud C., Hassanin A. (2013). Molecular systematics and phylogeography of the tribe Myonycterini (Mammalia, Pteropodidae) inferred from mitochondrial and nuclear markers. Mol. Phylogenet. Evol..

[50] Rambaut A., Drummond A.J., Xie D., Baele G., Suchard M.A. (2018). Posterior Summarization in Bayesian Phylogenetics Using Tracer 1.7. Syst. Biol..

[51] Mao X.G., Zhu G.J., Zhang S., Rossiter S.J. (2010). Pleistocene climatic cycling drives intra-specific diversification in the intermediate horseshoe bat (*Rhinolophus affinis*) in Southern China. Mol. Ecol..

[52] Tajima F. (1989). Statistical method for testing the neutral mutation hypothesis by DNA polymorphism. Genetics.

[53] Fu Y.X. (1997). Statistical tests of neutrality of mutations against population growth, hitchhiking and background selection. Genetics.

[54] Ramos-Onsins S.E., Rozas J. (2002). Statistical properties of new neutrality tests against population growth. Mol. Biol. Evol..

[55] Lin A.-Q., Csorba G., Li L.-F., Jiang T.-L., Lu G.-J., Thong V.D., Soisook P., Sun K.-P., Feng J., Emerson B. (2014). Phylogeography ofHipposideros armiger(Chiroptera: Hipposideridae) in the Oriental Region: The contribution of multiple Pleistocene glacial refugia and intrinsic factors to contemporary population genetic structure. J. Biogeogr..

[56] Yu Y., Harris A.J., Blair C., He X. (2015). RASP (Reconstruct Ancestral State in Phylogenies): A tool for historical biogeography. Mol. Phylogenet. Evol..

[57] Phillips S.J., Anderson R.P., Schapire R.E. (2006). Maximum entropy modeling of species geographic distributions. Ecol. Model..

[58] Hijmans R.J., Graham C.H. (2006). The ability of climate envelope models to predict the effect of climate change on species distributions. Glob. Change Biol..

[59] Qiu Y.X., Fu C.X., Comes H.P. (2011). Plant molecular phylogeography in China and adjacent regions: Tracing the genetic imprints of Quaternary climate and environmental change in the world’s most diverse temperate flora. Mol. Phylogenet. Evol..

[60] Meng X., Liu T., Zhang L., Jin L., Sun K., Feng J. (2021). Effects of colonization, geography and environment on genetic divergence in the intermediate leaf-nosed bat, *Hipposideros larvatus*. Animals.

[61] Shimada T., Aplin K.P., Jogahara T., Lin L.-K., Herbreteau V., Gonzalez J.-P., Suzuki H. (2007). Complex phylogeographic structuring in a continental small mammal from East Asia, the rice field mouse, *Mus caroli* (Rodentia, Muridae). Mamm. Study.

[62] Lv X., Cheng J., Meng Y., Chang Y., Xia L., Wen Z., Ge D., Liu S., Yang Q. (2018). Disjunct distribution and distinct intraspecific diversification of *Eothenomys melanogaster* in South China. BMC Evol. Biol..

[63] Toews D.P.L., Brelsford A. (2012). The biogeography of mitochondrial and nuclear discordance in animals. Mol. Ecol..

[64] Padial J.M., Miralles A., De la Riva I., Vences M. (2010). The integrative future of taxonomy. Front. Zool..

[65] Dayrat B. (2005). Towards integrative taxonomy. Biol. J. Linn. Soc..

[66] Sukumaran J., Knowles L.L. (2017). Multispecies coalescent delimits structure, not species. Proc. Natl. Acad. Sci. USA.

[67] Chornelia A., Lu J.M., Hughes A.C. (2022). How to Accurately Delineate Morphologically Conserved Taxa and Diagnose Their Phenotypic Disparities: Species Delimitation in Cryptic Rhinolophidae (Chiroptera). Front. Ecol. Evol..

