# Supplementary material for: Evolutionary History of the Rhinolophus pusillus Species Complex Reveals Phylogeographic Diversification Across the East Asian Mainland–Island System
Source: Animals (Basel). 2026 Aug 25;16(17):2669. doi: 10.3390/ani16172669 (PMC13564303; doi:10.3390/ani16172669)
Supplement: Supplementary file 1 [file animals-16-02669-s001.zip › Figure S1.pdf]

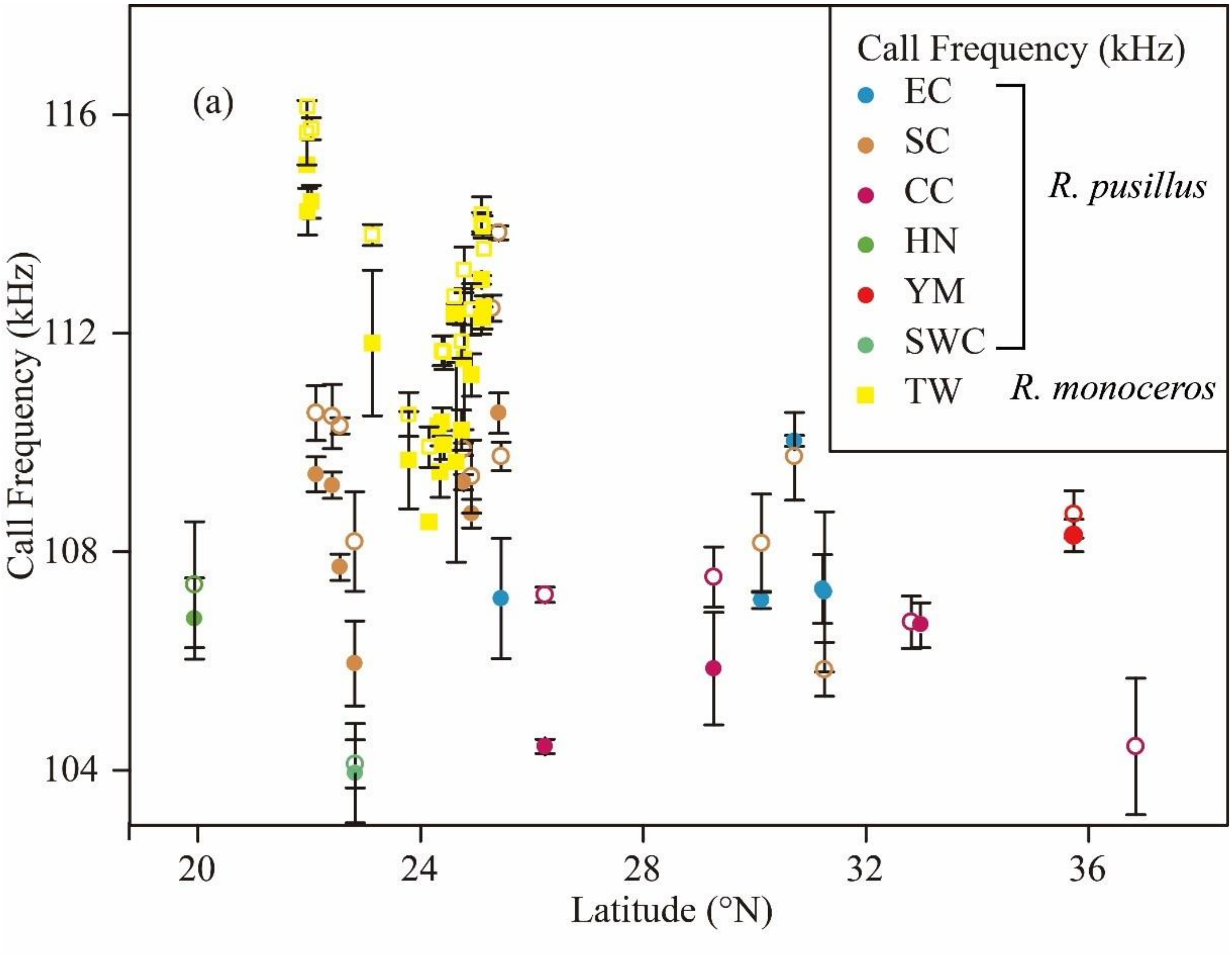

(a)
Call Frequency (kHz)
EC
SC
CC
HN
YM
SWC
TW
R. pusillus
R. monoceros
116
112
108
104
20
24
28
32
36
Latitude (°N)

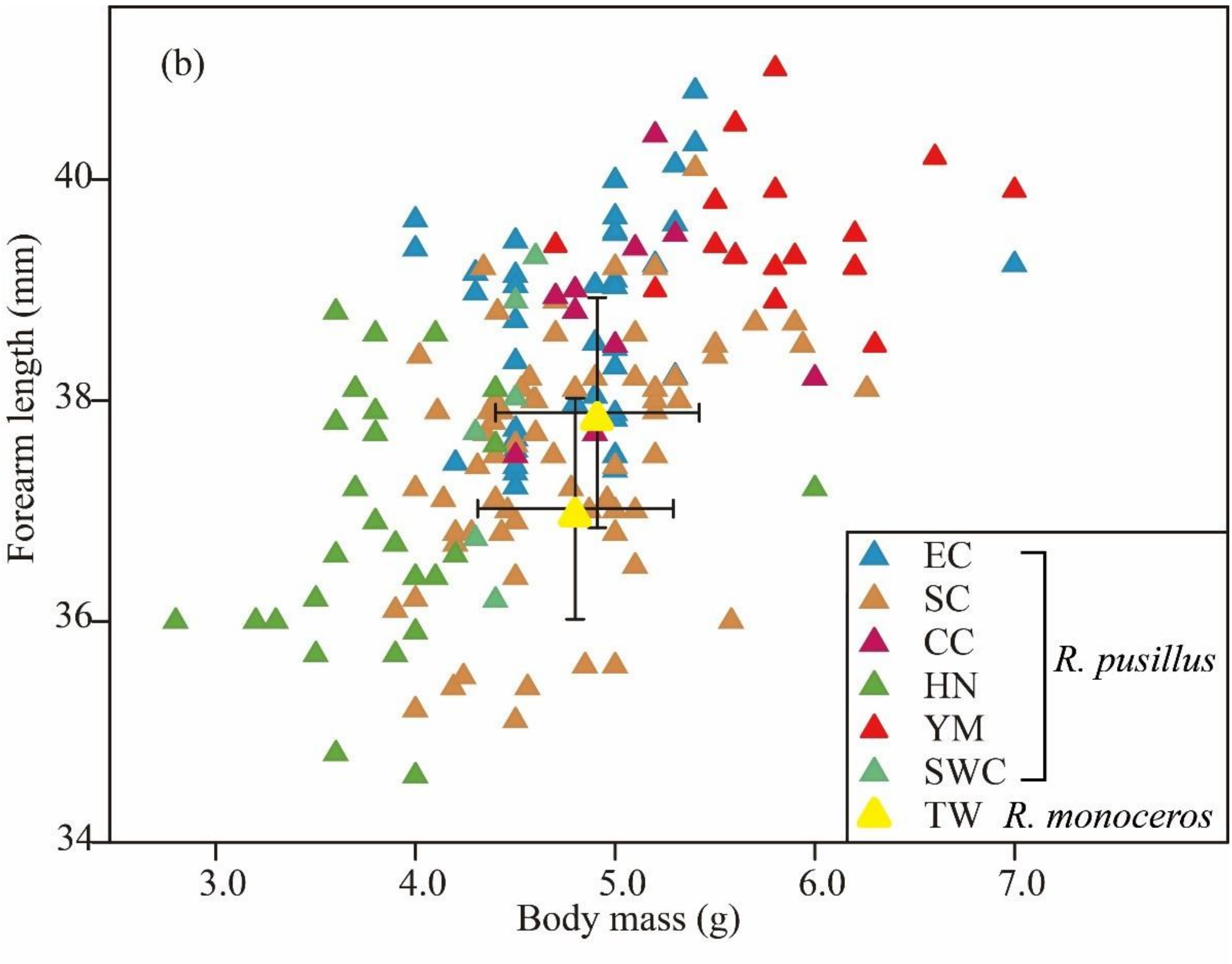

(b)
Forearm length (mm)
40
38
36
34
3.0
4.0
5.0
6.0
7.0
Body mass (g)
EC
SC
CC
HN
YM
SWC
TW
R. pusillus
R. monoceros

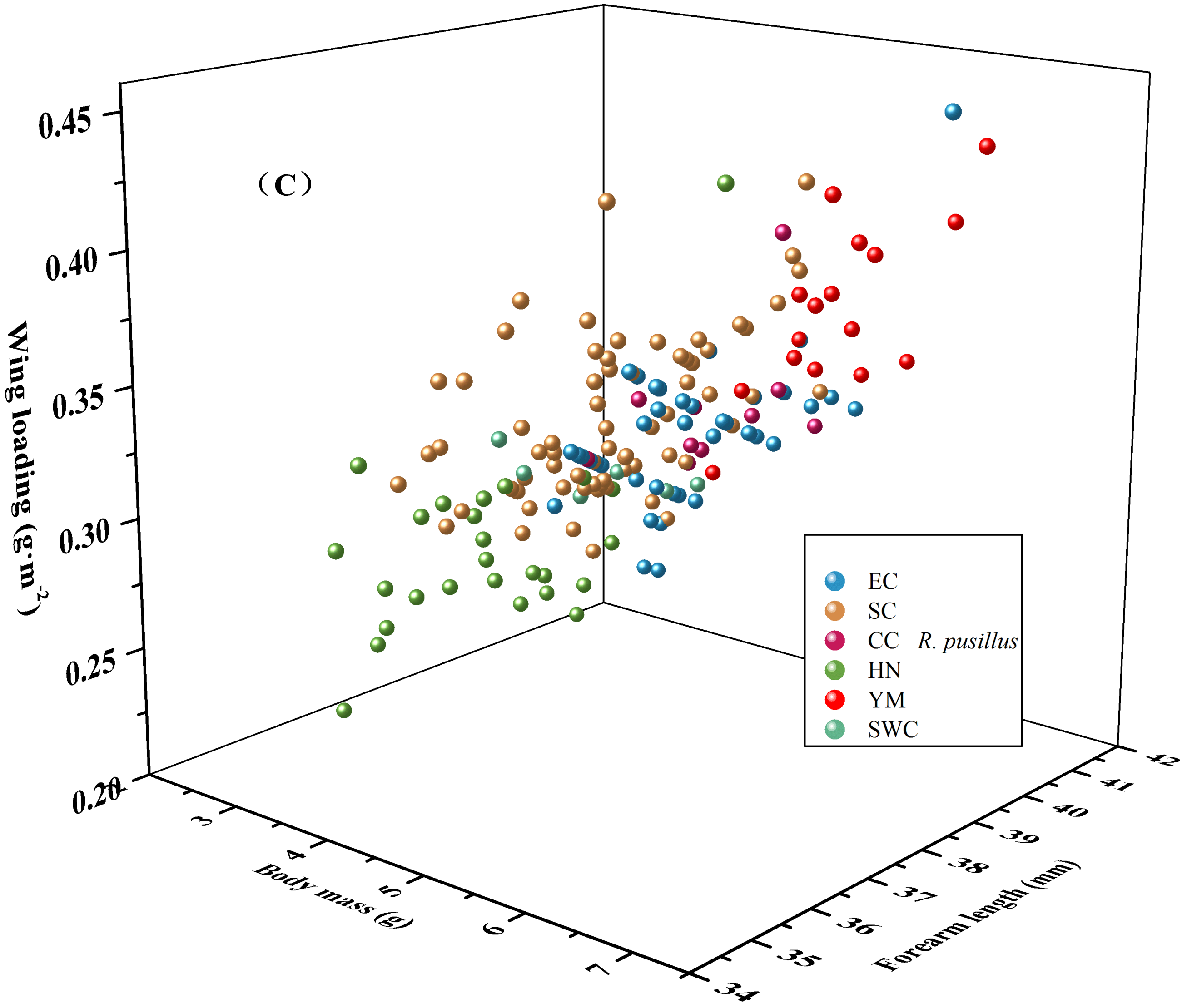
(C)
Wing loading (g·m^{-2})
0.45
0.40
0.35
0.30
0.25
0.20
Body mass (g)
3
4
5
6
7
Forearm length (mm)
34
35
36
37
38
39
40
41
42
EC
SC
CC
HN
YM
SWC
R. pusillus
