# Supplementary material for: Evolutionary History of the Rhinolophus pusillus Species Complex Reveals Phylogeographic Diversification Across the East Asian Mainland–Island System
Source: Animals (Basel). 2026 Aug 25;16(17):2669. doi: 10.3390/ani16172669 (PMC13564303; doi:10.3390/ani16172669)
Supplement: Supplementary file 1 [file animals-16-02669-s001.zip › Figure S2.pdf]

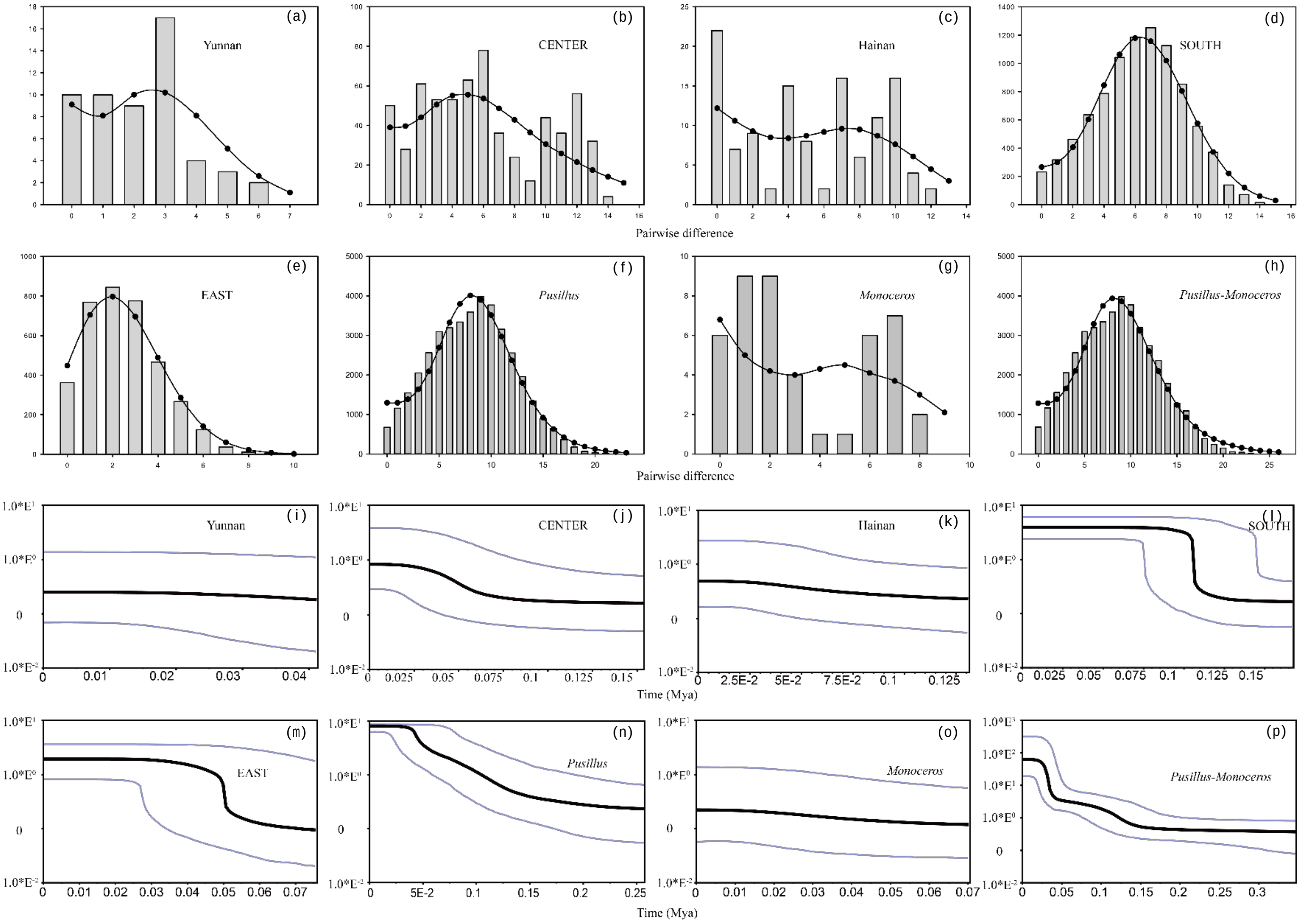

(a)
Yunnan
(b)
CENTER
(c)
Hainan
(d)
SOUTH
Pairwise difference
(e)
EAST
(f)
Pusillus
(g)
Monoceros
(h)
Pusillus-Monoceros
Pairwise difference
(i)
Yunnan
(j)
CENTER
(k)
Hainan
(l)
SOUTH
Time (Mya)
(m)
EAST
(n)
Pusillus
(o)
Monoceros
(p)
Pusillus-Monoceros
Time (Mya)
