# Supplementary material for: Evolutionary History of the Rhinolophus pusillus Species Complex Reveals Phylogeographic Diversification Across the East Asian Mainland–Island System
Source: Animals (Basel). 2026 Aug 25;16(17):2669. doi: 10.3390/ani16172669 (PMC13564303; doi:10.3390/ani16172669)
Supplement: Supplementary file 1 [file animals-16-02669-s001.zip › Figure_S3_STRUCTURE.pdf]

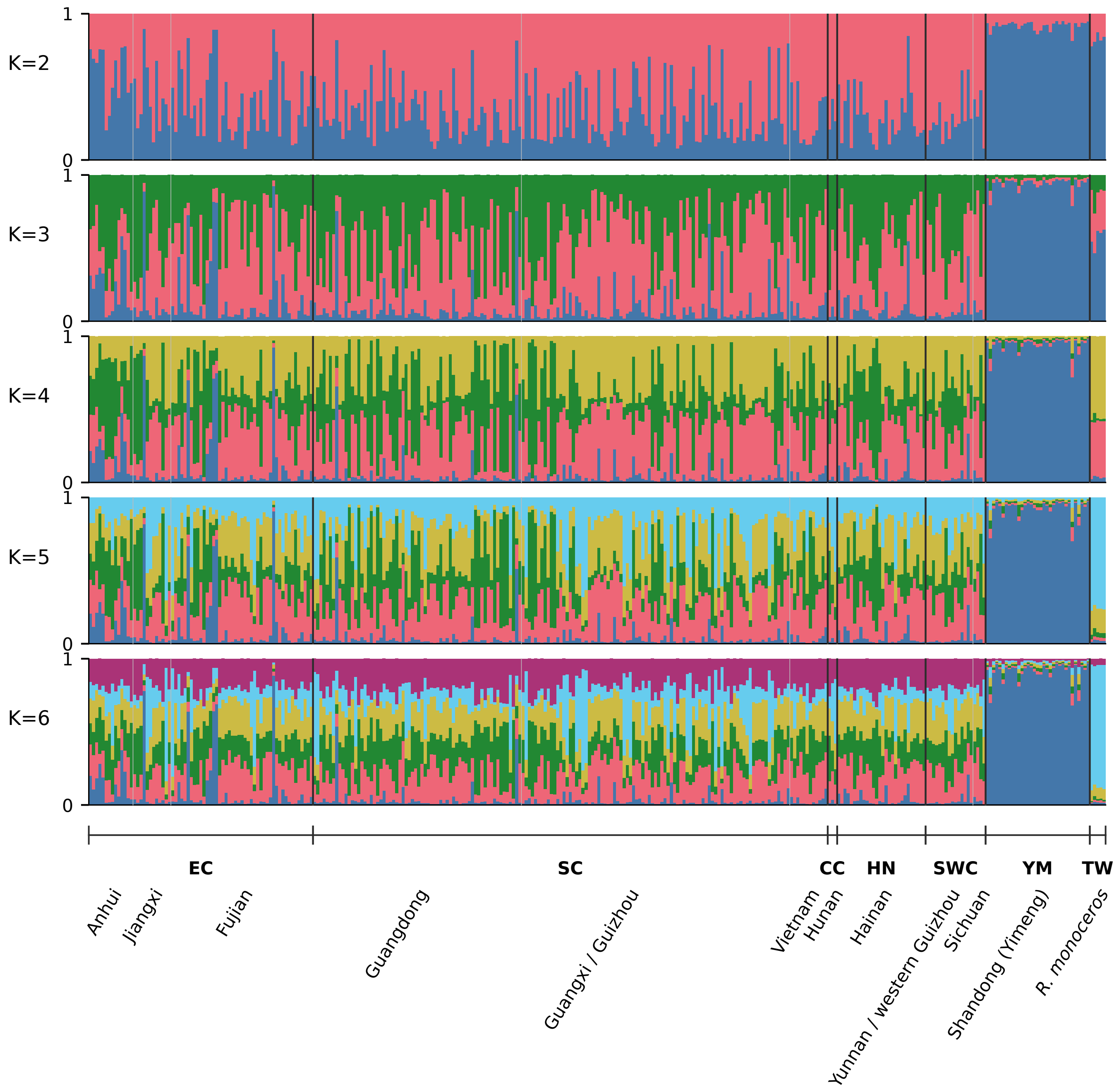

K=2
K=3
K=4
K=5
K=6
1
0
EC
SC
CC
HN
SWC
YM
TW
Anhui
Jiangxi
Fujian
Guangdong
Guangxi / Guizhou
Vietnam
Hunan
Hainan
Yunnan / western Guizhou
Sichuan
Shandong (Yimeng)
R. monoceros
